# Supplementary material for: Patient preferences for Remote cochlear implant management: A discrete choice experiment
Source: PLoS One. 2025 Jun 3;20(6):e0320421. doi: 10.1371/journal.pone.0320421 (PMC12133006; doi:10.1371/journal.pone.0320421)

**Figure S1**: Thematic analysis of focus group discussions with adult CI users (combine outcomes from one group with Remote Check experience [n=5] and one group with no Remote Check experience[n=5])


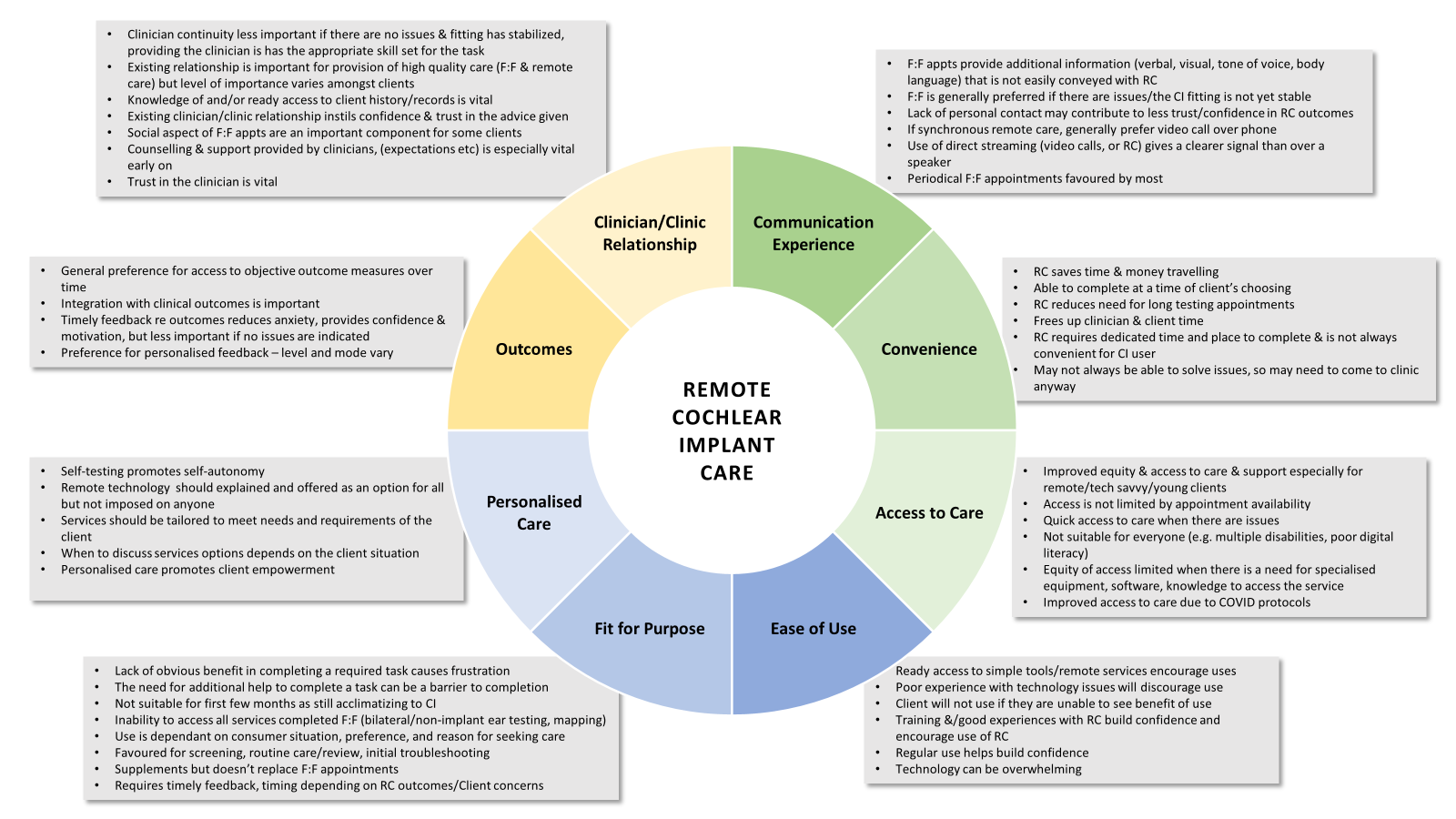

Supplement: S1 Fig — (DOCX) [file pone.0320421.s001.docx]
