# Supplementary material for: Patient preferences for Remote cochlear implant management: A discrete choice experiment
Source: PLoS One. 2025 Jun 3;20(6):e0320421. doi: 10.1371/journal.pone.0320421 (PMC12133006; doi:10.1371/journal.pone.0320421)
Supplement: S1 Table — (DOCX) [file pone.0320421.s002.docx]

**Table S1: Statistical analysis of demographic factors associated with willingness to accept a future remote check appointment in various formats.**

|  | **Participants who would accept (A) or refuse (R) a future remote check appointment in the following format** | | | | |
| --- | --- | --- | --- | --- | --- |
| **Demographic factor** | **Trouble shooting appointment** | **Additional monitoring (1^st^ 6 months post-switch on)** | **Completion of speech and hearing parts of annual review** | **Long-term monitoring** | **Only in-clinic appointments accepted** |
| **Age (years)**  (n, mean ±sd)^1^  (n, median; (25%-75%)^2^ | ^1`^A: (90, 61.6±12.2)  R: (33, 68.0±13.2)  *t* =-2.6, DF=121  ***(p=0.012)*** | ^2^A: (n=46) 60.0; (51.8-67.3)  R: (n= 77, 68.0; (57.0-76.0)13.2)  U(46, 77)=1190.5  ***(p=0.002)*** | ^1^A: (78, 60.56±12.5)  R: (45, 68.0±11.9)  *t* =-3.2, DF=121  ***(p=0.002)*** | ^2^A: (n=81) 63.0; (53.0-70.0)  R: (n= 42, 68.5; (59.5-77.0)13.2)  U(81,42)=1355.0  (p=0.065) | ^1^A: (109, 62.2±12.7)  R: (15, 71.5±9.9)  *t* =-2.7, DF=122  ***(p=0.007)*** |
| **Gender**  (Male:Female)^3^ | A: 56:34  R: 20: 13  X^2^(DF=1, n=123) = 0.00211  (p=0.963) | A: 26:50  R: 20: 27  X^2^(DF=1, n=123) = 0.544  (p=0.461) | A: 47:29  R: 31:16  X^2^(DF=1, n=123) = 0.0717  (p=0.789) | A: 49:27  R: 32:15  X^2^(DF=1, n=123) = 0.0461  (p=0.830) | A: 70:6  R: 39:8  X^2^(DF=1, n=123) = 1.579  (p=0.209) |
| **Annual household Income (AUD)***  **(**0): <$25000  (1): $25,000-$49,999  (2): $50,000-$69,999  (3): $70,000-$99,999  (4): $100,000-$124,999  (5): $125,000-$149,999  (6): $150,000 or more  Prefer not to answer (n=27) | A: (n=90)  Median= (3) $70,000-$99,999  25%= (1) $25,000-$49,000  75%= (4.75) $100,000-$149,999  R: (n=33)  Median=(2) $50,000-$69,999  25%= (0) <$25,000  75%= (4) $100,000-$124,999  U(81,42)=676.5  ***(p=0.035)*** | A: (n=46)  Median= (3) $70,000-$99,999  25%= (1) $25,000-$49,000  75%= (4.25) $100,000-$149,999  R: (n=77)  Median=(2) $50,000-$69,999  25%= (1) $25,000-$49,999  75%= (4) $100,000-$124,999  U(81,42)=925.5  (p=0.221) | A: (n=78)  Median= (3) $70,000-$99,999  25%= (1) $25,000-$49,000  75%= (4.25) $100,000-$149,999  R: (n=45)  Median=(2) $50,000-$69,999  25%= (1) $25,000-$49,999  75%= (4) $100,000-$124,999  U(81,42)=936.0  (p=0.213) | A: (n=81)  Median=(3) $70,000-$99,999  25%= (1) $25,000-$49,000  75%= (4) $100,000-$124,999  R: (n=42)  Median=(2) $50,000-$69,999  25%= (1) $25,000-$49,999  75%= (4) $100,000-$124,999  U(81,42)=899.5  (p=0.189) | A: (n=109)  Median=(3) $70,000-$99,999  25%= (1) $25,000-$49,000  75%= (4) $100,000-$124,999  R: (n=14)  Median=(1) $25,000-$49,999  25%= (0.25) <25,000  75%= (3.5) $100,000-$149,999  U(81,42)=339.5  (p=0.054) |
| **Satisfaction with CI*:**  Q:I am satisfied with my CI outcomes   1. Strongly disagree 2. Somewhat disagree 3. Neither agree nor disagree 4. Somewhat agree 5. Strongly agree | A: (n=90)  Median= (4) strongly agree  25%= (3) somewhat agree  75%= (4) strongly agree  R: (n=33)  Median= (4) strongly agree  25%= (3) somewhat agree  75%= (4) strongly agree  U(81,42)=1384.0  (p=0.479) | A: (n=46)  Median= (4) strongly agree  25%= (3) somewhat agree  75%= (4) strongly agree  R: (n=77)  Median= (4) strongly agree  25%= (3) somewhat agree  75%= (4) strongly agree  U(81,42)=1642.0  (p=0.407) | A: (n=78)  Median= (4) strongly agree  25%= (3) somewhat agree  75%= (4) strongly agree  R: (n=45)  Median= (4) strongly agree  25%= (3) somewhat agree  75%= (4) strongly agree  U(81,42)=1746.0  (p=0.956) | A: (n=81)  Median= (4) strongly agree  25%= (3.5) somewhat-strongly agree  75%= (4) strongly agree  R: (n=42)  Median= (4) strongly agree  25%= (3) somewhat agree  75%= (4) strongly agree  U(81,42)=1426.0  (p=0.071) | A: (n=109)  Median= (4) strongly agree  25%= (3) somewhat agree  75%= (4) strongly agree  R: (n=15)  Median=(3.5) somewhat-strongly agree  25%= (3) somewhat agree  75%= (4) strongly agree  U(81,42)=588.5  (p=0.087) |
| **Out of pocket cost for in-clinic appointment (AUD)** | A: (n=90)  Median= $50  25%= $10  75%= $150  R: (n=33)  Median= $25  25%= $5  75%= $115  U(81,42)=1150.0  (p=0.169) | A: (n=46)  Median= $25  25%= $10  75%= $135  R: (n=77)  Median= $50  25%= $10  75%= $120  U(81,42)=1469.5  (p=0.238) | A: (n=78)  Median= $30  25%= $10  75%= $137.5  R: (n=45)  Median= $50  25%= $15  75%= $120  U(81,42)=1595  (p=0.676) | A: (n=81)  Median= $40  25%= $10  75%= $130  R: (n=42)  Median= $47.5  25%= $11.25  75%= $120  U(81,42)=1561.0  (p=0.830) | A: (n=109)  Median = $50  25%= $10  75%= $130  R: (n=15)  Median= $45  25%= $15  75%= $160  U(81,42)=657.5  (p=0.751) |
| **Able to make time for appointments***   1. Strongly disagree 2. Disagree 3. Niether agree nor disagree 4. Agree 5. Strongly agree | A: (n=90)  Median= (3) agree  25%= (3) agree  75%= (4) strongly agree  R: (n=33)  Median= (4) strongly agree  25%= (3) agree  75%= (4) strongly agree  U(81,42)=1085.0  (p=0.059) | A: (n=46)  Median= (3) agree  25%= (2) neither agree nor disagree  75%= (4) strongly agree  R: (n=77)  Median= (3) agree  25%= (3) agree  75%= (4) strongly agree  U(81,42)=1384.5  (p=0.079) | A: (n=78)  Median= (3) agree  25%= (3) agree  75%= (4) strongly agree  R: (n=45)  Median= (3) agree  25%= (3) agree  75%= (4) strongly agree  U(81,42)=1622.0  (p=0.773) | A: (n=81)  Median= (3) agree  25%= (2) neither agree nor disagree  75%= (4) strongly agree  R: (n=42)  Median= (3) agree  25%= (3) agree  75%= (4) strongly agree  U(81,42)=1258.0  ***(p=0.042)*** | A: (n=109)  Median= (3) agree  25%= (3) agree  75%= (4) strongly agree  R: (n=15)  Median= (3) agree  25%= (3) agree  75%= (4) strongly agree  U(81,42)=623.5  (p=0.518) |
| **Device use (1^st^ implant)***   1. ½ year-<1 year 2. 1 year -<2 years 3. 2 years-<5 years 4. 5 years-<10 years 5. 10 years-<20 years 6. >20 years | A: (n=90)  Median= (2.5) 2-<10 years  25%= (2) 2-<5 years  75%= (3.25) 5-<20 years  R: (n=33)  Median = (3) 5-<10 years  25%= (2) 2-<5 years  75%= (4) 10-<20 years  U(81,42)=1343.5  (p=0.402) | A: (n=46)  Median = (3) 5-<10 years  25%= (2) 2-<5 years  75%= (4) 10-<20 years  R: (n=77)  Median = (3) 5-<10 years  25%= (2) 2-<5 years  75%= (3) 5-<10 years  U(81,42)=1617.5  (p=0.405) | A: (n=78)  Median = (3) 5-<10 years  25%= (2) 2-<5 years  75%= (3.25) 5-<20 years  R: (n=45)  Median = (3) 5-<10 years  25%= (2) 2-<5 years  75%= (4) 10-<20 years  U(81,42)=1714.5  (p=0.827) | A: (n=81)  Median = (3) 5-<10 years  25%= (2) 2-<5 years  75%= (4) 10-<20 years  R: (n=42)  Median = (3) 5-<10 years  25%= (2) 2-<5 years  75%= (3) 5-<10 years  U(81,42)=1700.0  (p=0.998) | A: (n=109)  Median = (3) 5-<10 years  25%= (2) 2-<5 years  75%= (4) 10-<20 years  R: (n=15)  Median = (3) 5-<10 years  25%= (2) 2-<5 years  75%= (3.25) 5-<20 years  U(81,42)=692.0  (p=0.559) |
| **Device configuration**  CIHA(A)/UniCI(A)/UniCI(B) /CIHA(B)/BilatCI | A: 18:17:12:20:23  R: 5:2:5:4:17  X^2^(DF=4, n=123) = 9.200 (p=0.056) | A: 11:7:5:10:13  R: 12:12:12:14:27  X^2^(DF=4, n=123) = 2.131 (p=0.712) | A: 16:14:11:13:24  R: 7:5:6:11:16  X^2^(DF=4, n=123) = 2.337 (p=0.674) | A: 17:14:11:11:28  R: 6:5:6:13:12  X^2^(DF=4, n=123) = 5.776 (p=0.217) | ^4^A: 21:18:16:21:33  R: 2:1:1:3:7  X^2^(DF=4, n=123) = 2.894 (p=0.576) |

Statistical analysis of demographic factors associated with willingness to accept a remote check review appointment in various formats are shown. Analysis is a combination of ^1^t-test, where normality tests were passed, ^2^Mann-Whitney rank sum tests, where normality tests failed or categorical data collected, ^3^Chi^2^test when categorical data could not be ranked. ^4^It is noted that the in-clinic only category of review for device configuration has only very limited numbers per device configuration, thus the accuracy of the test is limited. Codes for categorical data used for analysis are shown in brackets at the front of each category where the demographic factor is marked with an *. For device configuration categories, abbreviations are definded as following; CIHA(A) – CI one ear, HA other ear (poor hearing with HA), UniCI(A) -CI one ear, other ear no HA (good hearing), UniCI(B) – CI one ear, other ear no HA (poor hearing), CIHA(B) – CI one ear, HA other ear (good hearing with HA), BilatCI – CIs in both ears.
