# Supplementary material for: Patient preferences for Remote cochlear implant management: A discrete choice experiment
Source: PLoS One. 2025 Jun 3;20(6):e0320421. doi: 10.1371/journal.pone.0320421 (PMC12133006; doi:10.1371/journal.pone.0320421)
Supplement: S2 Table — (DOCX) [file pone.0320421.s003.docx]

**Table S2**: DCE results – pooled and per scenario (Conditional fixed-effects logistic regression)

| **Attributes** | **Levels** | **Pooled** | | | **Troubleshooting scenario** | | | **Long-term review scenario** | | | **Acute care scenario** | | |
| --- | --- | --- | --- | --- | --- | --- | --- | --- | --- | --- | --- | --- | --- |
|  |  | **Co-efficient** | **p-value** | **95% CI** | **Co-efficient** | **p-value** | **95% CI** | **Co-efficient** | **p-value** | **95% CI** | **Co-efficient** | **p-value** | **95% CI** |
| Who reviews Remote Check test | Reference level: Trained administration staff | | | | | | | | | | | | |
|  | Any trained audiologist | 0.325 | 0.001 | 0.141; 0.509 | 0.105 | 0.488 | -0.191, 0.401 | 0.707 | <0.001 | 0.420, 0.994 | 0.209 | 0.153 | -0.077, 0.494 |
|  | My regular implant audiologist | 0.683 | <0.001 | 0.469; 0.898 | 0.763 | <0.001 | 0.448, 1.078 | 0.801 | <0.001 | 0.480, 1.123 | 0.545 | 0.001 | 0.229, 0.860 |
| Information provided regarding outcome | Reference level: Response only if test indicates a problem | | | | | | | | | | | | |
|  | Response indicating good / bad result and next steps | 0.062 | 0.461 | -0.103; 0.228 | -0.048 | 0.742 | -0.334, 0.238 | 0.227 | 0.135 | -0.071, 0.526 | 0.031 | 0.836 | -0.260, 0.321 |
|  | Detailed comparison with previous results | 0.199 | 0.037 | 0.012; 0.387 | 0.077 | 0.598 | -0.210, 0.364 | 0.344 | 0.027 | 0.039, 0.648 | 0.188 | 0.204 | -0.102, 0.479 |
| Timing of feedback | Reference level: The next day | | | | | | | | | | | | |
|  | Within a week | -0.062 | 0.452 | -0.223; 0.099 | -0.051 | 0.724 | -0.332, 0.230 | -0.167 | 0.293 | -0.478, 0.144 | 0.001 | 0.996 | -0.299, 0.301 |
|  | Within two weeks | -0.300 | 0.001 | -0.473; -0.128 | -0.463 | 0.002 | -0.751, -0.175 | -0.243 | 0.105 | -0.537, 0.051 | -0.221 | 0.115 | -0.495, 0.053 |
| How outcome information is received | Reference level: Nucleus Smart App notification | | | | | | | | | | | | |
|  | Emailed report | -0.019 | 0.845 | -0.216; 0.177 | 0.022 | 0.876 | -0.258, 0.302 | -0.123 | 0.466 | -0.453, 0.207 | 0.012 | 0.946 | -0.342, 0.367 |
|  | Videoconference/phone call with my audiologist | -0.195 | 0.053 | -0.392; 0.002 | -0.051 | 0.761 | -0.382, 0.279 | -0.091 | 0.572 | -0.409, 0.226 | -0.459 | 0.004 | -0.767, -0.150 |
|  | Face-to-face meeting with my audiologist | -0.256 | 0.022 | -0.476; -0.036 | -0.242 | 0.161 | -0.580, 0.097 | -0.262 | 0.115 | -0.588, 0.064 | -0.304 | 0.077 | -0.642, 0.033 |
| Cost to access services | Reference level: $10 per review | | | | | | | | | | | | |
|  | $30 per review | -0.490 | <0.001 | -0.686; -0.294 | -0.654 | <0.001 | -0.976, -0.333 | -0.386 | 0.023 | -0.719, -0.053 | -0.444 | 0.005 | -0.754, -0.133 |
|  | $40 annual fee (unlimited checks) | -0.242 | 0.035 | -0.467; -0.017 | -0.292 | 0.08 | -0.619, 0.035 | -0.326 | 0.057 | -0.662, 0.009 | -0.149 | 0.386 | -0.487, 0.188 |
|  | $120 annual fee (unlimited checks) | -1.042 | <0.001 | -1.316; -0.768 | -1.029 | <0.001 | -1.405, -0.652 | -1.24 | <0.001 | -1.632, -0.848 | -0.909 | <0.001 | -1.296, -0.522 |

CI: confidence interval; DCE: discrete choice experiment

Troubleshooting scenario: Imagine you are having a problem with your cochlear implant and it is suggested that you could complete a Remote Check test to test your cochlear implant function at home instead of waiting for a clinical appointment.

Long-term review scenario: Imagine you are due for your yearly cochlear implant review and it is suggested that you complete a Remote Check test of your cochlear implant function at home. An optional, short "in-clinic" appointment is available after your Remote Check if it is required.

Acute care: Imagine you have recently got your cochlear implant. You aren’t due for another clinical appointment for a while, or you can’t make it to your next appointment, but you would like reassurance that things are progressing between appointments. You are offered a Remote Check test.
