# Supplementary material for: Patient preferences for Remote cochlear implant management: A discrete choice experiment
Source: PLoS One. 2025 Jun 3;20(6):e0320421. doi: 10.1371/journal.pone.0320421 (PMC12133006; doi:10.1371/journal.pone.0320421)
Supplement: S3 Table — Choice pairs were identified using Ngene software. Scenario 1= troubleshooting, Scenario 2=long-term review appointment, Scenario 3=acute care. Attribute levels are listed in table 1 of the main manuscript. For example, Choice set 1 presents a choice pair of the following: OPTION A: reviewed by regular audiologist, provided with a detailed comparison of hearing and speech tests with previous results, feedback provided within a week, information received via a notification in the Nucleus Smart app, at a cost of $120 annual fee for unlimited number of checks. OPTION B: reviewed by a trained admin staff, with a response only if the test indicated a problem, with information provided by the next day, via a face-to-face meeting with the audiologist, at an annual fee of $40 for unlimited checks. (DOCX) [file pone.0320421.s004.docx]

Table S3: DCE design. Choice pairs were identified using Ngene software. Scenario 1= troubleshooting, Scenario 2=long-term review appointment, Scenario 3=acute care. Attribute levels are listed in table 1 of the main manuscript. For example, Choice set 1 presents a choice pair of the following: OPTION A: reviewed by regular audiologist, provided with a detailed comparison of hearing and speech tests with previous results, feedback provided within a week, information received via a notification in the Nucleus Smart app, at a cost of $120 annual fee for unlimited number of checks. OPTION B: reviewed by a trained admin staff, with a response only if the test indicated a problem, with information provided by the next day, via a face-to-face meeting with the audiologist, at an annual fee of $40 for unlimited checks.

|  | **CHOICE PAIRS - (allocated levels for attributes for alternative 1 and alternative 2)** | | | | | | | | | |  |  |  |
| --- | --- | --- | --- | --- | --- | --- | --- | --- | --- | --- | --- | --- | --- |
| **choice set** | **alt1.who** | **alt1.info** | **alt1.timing** | **alt1.notification** | **alt1.cost** | **alt2.who** | **alt2.info** | **alt2.timing** | **alt2.notification** | **alt2.cost** | **Block # with scenario** | **3 Scenarios** | **Question ID (sc,block, choice set)** |
| 1 | 2 | 2 | 1 | 0 | 3 | 0 | 0 | 0 | 3 | 2 | 1 | 1 | 101001 |
| 2 | 1 | 0 | 2 | 2 | 0 | 2 | 2 | 0 | 0 | 1 | 1 | 1 | 101002 |
| 3 | 0 | 0 | 0 | 3 | 1 | 2 | 1 | 1 | 1 | 3 | 1 | 1 | 101003 |
| 4 | 1 | 0 | 1 | 0 | 3 | 2 | 2 | 2 | 2 | 2 | 1 | 1 | 101004 |
| 5 | 0 | 1 | 0 | 1 | 3 | 1 | 2 | 1 | 2 | 1 | 1 | 2 | 201005 |
| 6 | 1 | 1 | 2 | 3 | 2 | 2 | 0 | 0 | 2 | 1 | 1 | 2 | 201006 |
| 7 | 2 | 1 | 0 | 2 | 3 | 1 | 2 | 1 | 3 | 0 | 1 | 2 | 201007 |
| 8 | 1 | 2 | 1 | 0 | 3 | 2 | 1 | 2 | 1 | 0 | 1 | 2 | 201008 |
| 9 | 1 | 1 | 2 | 3 | 0 | 0 | 2 | 0 | 1 | 2 | 1 | 3 | 301009 |
| 10 | 2 | 0 | 0 | 3 | 1 | 1 | 2 | 1 | 1 | 3 | 1 | 3 | 301010 |
| 11 | 0 | 1 | 1 | 1 | 1 | 2 | 0 | 2 | 0 | 0 | 1 | 3 | 301011 |
| 12 | 1 | 2 | 2 | 1 | 3 | 0 | 1 | 1 | 2 | 2 | 1 | 3 | 301012 |
| 13 | 1 | 2 | 0 | 1 | 2 | 2 | 1 | 1 | 2 | 0 | 2 | 1 | 102013 |
| 14 | 1 | 1 | 1 | 0 | 0 | 0 | 0 | 2 | 1 | 3 | 2 | 1 | 102014 |
| 15 | 1 | 1 | 2 | 2 | 1 | 0 | 2 | 1 | 0 | 3 | 2 | 1 | 102015 |
| 16 | 1 | 2 | 2 | 2 | 0 | 2 | 0 | 1 | 0 | 2 | 2 | 1 | 102016 |
| 17 | 1 | 0 | 1 | 1 | 0 | 0 | 2 | 2 | 3 | 3 | 2 | 2 | 202017 |
| 18 | 0 | 1 | 2 | 1 | 0 | 2 | 0 | 0 | 2 | 3 | 2 | 2 | 202018 |
| 19 | 0 | 0 | 2 | 0 | 2 | 2 | 1 | 0 | 1 | 0 | 2 | 2 | 202019 |
| 20 | 2 | 1 | 1 | 2 | 2 | 1 | 0 | 2 | 3 | 0 | 2 | 2 | 202020 |
| 21 | 1 | 1 | 0 | 2 | 2 | 0 | 0 | 2 | 1 | 1 | 2 | 3 | 302021 |
| 22 | 0 | 0 | 1 | 3 | 0 | 1 | 1 | 2 | 0 | 1 | 2 | 3 | 302022 |
| 23 | 2 | 0 | 2 | 2 | 0 | 0 | 2 | 1 | 1 | 1 | 2 | 3 | 302023 |
| 24 | 1 | 1 | 0 | 0 | 2 | 2 | 0 | 1 | 1 | 0 | 2 | 3 | 302024 |
| 25 | 2 | 1 | 0 | 3 | 2 | 0 | 2 | 2 | 2 | 3 | 3 | 1 | 103025 |
| 26 | 2 | 1 | 2 | 0 | 0 | 0 | 0 | 1 | 3 | 1 | 3 | 1 | 103026 |
| 27 | 2 | 0 | 1 | 2 | 2 | 1 | 2 | 0 | 0 | 3 | 3 | 1 | 103027 |
| 28 | 0 | 2 | 2 | 0 | 3 | 2 | 0 | 0 | 3 | 0 | 3 | 1 | 103028 |
| 29 | 2 | 2 | 0 | 0 | 2 | 0 | 1 | 2 | 2 | 0 | 3 | 2 | 203029 |
| 30 | 2 | 2 | 2 | 1 | 0 | 0 | 1 | 1 | 0 | 2 | 3 | 2 | 203030 |
| 31 | 0 | 2 | 0 | 2 | 0 | 2 | 0 | 2 | 3 | 3 | 3 | 2 | 203031 |
| 32 | 2 | 2 | 1 | 1 | 3 | 1 | 1 | 0 | 2 | 1 | 3 | 2 | 203032 |
| 33 | 2 | 2 | 2 | 3 | 2 | 1 | 1 | 1 | 0 | 1 | 3 | 3 | 303033 |
| 34 | 0 | 0 | 0 | 2 | 0 | 1 | 2 | 1 | 3 | 1 | 3 | 3 | 303034 |
| 35 | 0 | 0 | 0 | 0 | 3 | 2 | 1 | 1 | 2 | 1 | 3 | 3 | 303035 |
| 36 | 2 | 2 | 0 | 2 | 3 | 0 | 1 | 1 | 3 | 2 | 3 | 3 | 303036 |
| 37 | 1 | 0 | 1 | 2 | 2 | 0 | 1 | 0 | 0 | 0 | 4 | 1 | 104037 |
| 38 | 2 | 0 | 1 | 0 | 2 | 0 | 2 | 0 | 3 | 0 | 4 | 1 | 104038 |
| 39 | 1 | 2 | 0 | 3 | 2 | 0 | 0 | 2 | 1 | 0 | 4 | 1 | 104039 |
| 40 | 0 | 2 | 0 | 3 | 0 | 2 | 1 | 2 | 0 | 3 | 4 | 1 | 104040 |
| 41 | 0 | 0 | 2 | 0 | 1 | 2 | 1 | 1 | 3 | 0 | 4 | 2 | 204041 |
| 42 | 0 | 0 | 2 | 0 | 2 | 1 | 2 | 0 | 2 | 3 | 4 | 2 | 204042 |
| 43 | 1 | 2 | 2 | 1 | 1 | 0 | 0 | 1 | 3 | 3 | 4 | 2 | 204043 |
| 44 | 0 | 2 | 1 | 2 | 1 | 2 | 0 | 2 | 0 | 3 | 4 | 2 | 204044 |
| 45 | 0 | 2 | 2 | 2 | 2 | 2 | 0 | 0 | 1 | 0 | 4 | 3 | 304045 |
| 46 | 0 | 0 | 1 | 3 | 2 | 1 | 1 | 0 | 2 | 1 | 4 | 3 | 304046 |
| 47 | 2 | 2 | 0 | 1 | 2 | 1 | 0 | 2 | 3 | 3 | 4 | 3 | 304047 |
| 48 | 0 | 1 | 0 | 2 | 2 | 2 | 2 | 2 | 1 | 1 | 4 | 3 | 304048 |
| 49 | 1 | 1 | 2 | 3 | 2 | 0 | 0 | 1 | 2 | 1 | 5 | 1 | 105049 |
| 50 | 1 | 1 | 2 | 2 | 1 | 2 | 2 | 0 | 1 | 2 | 5 | 1 | 105050 |
| 51 | 0 | 1 | 1 | 1 | 2 | 1 | 2 | 0 | 0 | 1 | 5 | 1 | 105051 |
| 52 | 0 | 1 | 2 | 3 | 3 | 1 | 0 | 0 | 2 | 1 | 5 | 1 | 105052 |
| 53 | 2 | 2 | 1 | 0 | 0 | 1 | 0 | 0 | 1 | 1 | 5 | 2 | 205053 |
| 54 | 0 | 0 | 1 | 3 | 1 | 1 | 2 | 0 | 1 | 2 | 5 | 2 | 205054 |
| 55 | 2 | 2 | 1 | 2 | 1 | 0 | 1 | 2 | 0 | 0 | 5 | 2 | 205055 |
| 56 | 0 | 0 | 2 | 1 | 3 | 1 | 1 | 0 | 0 | 0 | 5 | 2 | 205056 |
| 57 | 1 | 2 | 1 | 3 | 0 | 0 | 1 | 2 | 1 | 1 | 5 | 3 | 305057 |
| 58 | 0 | 1 | 0 | 0 | 0 | 1 | 0 | 2 | 3 | 2 | 5 | 3 | 305058 |
| 59 | 2 | 0 | 0 | 3 | 0 | 1 | 1 | 1 | 1 | 2 | 5 | 3 | 305059 |
| 60 | 1 | 0 | 2 | 2 | 3 | 2 | 2 | 0 | 0 | 0 | 5 | 3 | 305060 |
| 61 | 0 | 1 | 1 | 0 | 3 | 2 | 0 | 2 | 2 | 2 | 6 | 1 | 106061 |
| 62 | 2 | 2 | 2 | 3 | 0 | 0 | 1 | 0 | 0 | 1 | 6 | 1 | 106062 |
| 63 | 1 | 1 | 1 | 1 | 2 | 2 | 2 | 2 | 3 | 1 | 6 | 1 | 106063 |
| 64 | 2 | 2 | 2 | 0 | 1 | 1 | 0 | 0 | 2 | 3 | 6 | 1 | 106064 |
| 65 | 0 | 0 | 0 | 1 | 1 | 1 | 2 | 2 | 0 | 2 | 6 | 2 | 206065 |
| 66 | 0 | 0 | 0 | 0 | 2 | 1 | 1 | 2 | 1 | 3 | 6 | 2 | 206066 |
| 67 | 2 | 2 | 1 | 2 | 0 | 0 | 0 | 0 | 0 | 1 | 6 | 2 | 206067 |
| 68 | 1 | 0 | 2 | 3 | 1 | 2 | 2 | 0 | 1 | 2 | 6 | 2 | 206068 |
| 69 | 2 | 2 | 1 | 2 | 3 | 1 | 1 | 2 | 3 | 2 | 6 | 3 | 306069 |
| 70 | 0 | 2 | 1 | 1 | 0 | 1 | 0 | 0 | 3 | 1 | 6 | 3 | 306070 |
| 71 | 1 | 0 | 2 | 2 | 2 | 2 | 1 | 1 | 3 | 1 | 6 | 3 | 306071 |
| 72 | 1 | 0 | 0 | 3 | 3 | 0 | 2 | 2 | 0 | 0 | 6 | 3 | 306072 |
| 73 | 2 | 1 | 0 | 0 | 1 | 0 | 2 | 1 | 3 | 3 | 7 | 1 | 107073 |
| 74 | 1 | 1 | 0 | 1 | 1 | 0 | 2 | 2 | 2 | 0 | 7 | 1 | 107074 |
| 75 | 2 | 0 | 0 | 1 | 2 | 0 | 2 | 1 | 2 | 3 | 7 | 1 | 107075 |
| 76 | 2 | 0 | 0 | 3 | 0 | 1 | 2 | 1 | 1 | 3 | 7 | 1 | 107076 |
| 77 | 2 | 1 | 1 | 0 | 1 | 1 | 2 | 0 | 1 | 2 | 7 | 2 | 207077 |
| 78 | 1 | 1 | 1 | 1 | 3 | 0 | 2 | 2 | 2 | 1 | 7 | 2 | 207078 |
| 79 | 2 | 1 | 1 | 2 | 1 | 1 | 0 | 0 | 1 | 0 | 7 | 2 | 207079 |
| 80 | 0 | 2 | 2 | 1 | 0 | 2 | 0 | 1 | 2 | 2 | 7 | 2 | 207080 |
| 81 | 1 | 0 | 1 | 1 | 1 | 0 | 1 | 0 | 0 | 3 | 7 | 3 | 307081 |
| 82 | 2 | 0 | 2 | 1 | 1 | 1 | 1 | 1 | 3 | 3 | 7 | 3 | 307082 |
| 83 | 0 | 2 | 0 | 3 | 0 | 1 | 0 | 1 | 1 | 3 | 7 | 3 | 307083 |
| 84 | 2 | 1 | 0 | 3 | 3 | 1 | 0 | 1 | 1 | 0 | 7 | 3 | 307084 |
| 85 | 0 | 1 | 0 | 3 | 1 | 1 | 0 | 2 | 0 | 3 | 8 | 1 | 108085 |
| 86 | 1 | 2 | 0 | 1 | 1 | 2 | 1 | 1 | 2 | 3 | 8 | 1 | 108086 |
| 87 | 2 | 1 | 2 | 3 | 1 | 0 | 0 | 0 | 0 | 2 | 8 | 1 | 108087 |
| 88 | 1 | 1 | 2 | 3 | 3 | 0 | 0 | 1 | 2 | 0 | 8 | 1 | 108088 |
| 89 | 2 | 2 | 1 | 0 | 2 | 0 | 0 | 0 | 2 | 3 | 8 | 2 | 208089 |
| 90 | 1 | 2 | 0 | 0 | 1 | 0 | 0 | 1 | 1 | 2 | 8 | 2 | 208090 |
| 91 | 2 | 0 | 0 | 0 | 3 | 0 | 1 | 1 | 2 | 2 | 8 | 2 | 208091 |
| 92 | 1 | 0 | 0 | 2 | 3 | 0 | 1 | 2 | 3 | 1 | 8 | 2 | 208092 |
| 93 | 1 | 0 | 2 | 0 | 0 | 2 | 1 | 1 | 3 | 3 | 8 | 3 | 308093 |
| 94 | 2 | 0 | 2 | 0 | 3 | 0 | 2 | 0 | 2 | 1 | 8 | 3 | 308094 |
| 95 | 1 | 1 | 2 | 2 | 2 | 2 | 2 | 0 | 3 | 3 | 8 | 3 | 308095 |
| 96 | 0 | 1 | 0 | 1 | 3 | 1 | 2 | 2 | 0 | 2 | 8 | 3 | 308096 |
| 97 | 1 | 0 | 0 | 0 | 0 | 2 | 1 | 2 | 3 | 1 | 9 | 1 | 109097 |
| 98 | 1 | 2 | 1 | 3 | 1 | 2 | 1 | 0 | 1 | 2 | 9 | 1 | 109098 |
| 99 | 1 | 0 | 1 | 3 | 0 | 2 | 1 | 2 | 0 | 1 | 9 | 1 | 109099 |
| 100 | 2 | 1 | 2 | 3 | 2 | 0 | 0 | 1 | 0 | 1 | 9 | 1 | 109100 |
| 101 | 0 | 1 | 2 | 1 | 0 | 1 | 2 | 1 | 3 | 2 | 9 | 2 | 209101 |
| 102 | 1 | 2 | 1 | 1 | 1 | 0 | 1 | 0 | 3 | 2 | 9 | 2 | 209102 |
| 103 | 0 | 1 | 0 | 2 | 2 | 2 | 2 | 1 | 3 | 0 | 9 | 2 | 209103 |
| 104 | 0 | 2 | 0 | 2 | 3 | 1 | 0 | 1 | 3 | 2 | 9 | 2 | 209104 |
| 105 | 2 | 2 | 2 | 0 | 2 | 1 | 1 | 0 | 3 | 0 | 9 | 3 | 309105 |
| 106 | 2 | 2 | 0 | 2 | 3 | 1 | 1 | 2 | 0 | 0 | 9 | 3 | 309106 |
| 107 | 2 | 2 | 1 | 0 | 0 | 1 | 1 | 2 | 1 | 3 | 9 | 3 | 309107 |
| 108 | 0 | 0 | 2 | 0 | 1 | 1 | 2 | 0 | 1 | 0 | 9 | 3 | 309108 |
| 109 | 1 | 0 | 1 | 2 | 3 | 0 | 2 | 2 | 1 | 2 | 10 | 1 | 110109 |
| 110 | 0 | 1 | 1 | 3 | 3 | 2 | 0 | 2 | 2 | 2 | 10 | 1 | 110110 |
| 111 | 2 | 1 | 0 | 1 | 3 | 0 | 2 | 1 | 0 | 2 | 10 | 1 | 110111 |
| 112 | 1 | 1 | 1 | 1 | 0 | 2 | 2 | 2 | 2 | 3 | 10 | 1 | 110112 |
| 113 | 0 | 2 | 2 | 2 | 3 | 2 | 0 | 1 | 1 | 1 | 10 | 2 | 210113 |
| 114 | 1 | 2 | 1 | 1 | 1 | 2 | 1 | 2 | 2 | 3 | 10 | 2 | 210114 |
| 115 | 2 | 1 | 1 | 1 | 1 | 0 | 0 | 2 | 0 | 0 | 10 | 2 | 210115 |
| 116 | 0 | 0 | 0 | 3 | 3 | 2 | 1 | 2 | 2 | 0 | 10 | 2 | 210116 |
| 117 | 0 | 2 | 2 | 3 | 1 | 2 | 0 | 1 | 0 | 0 | 10 | 3 | 310117 |
| 118 | 0 | 1 | 1 | 2 | 0 | 2 | 2 | 0 | 3 | 2 | 10 | 3 | 310118 |
| 119 | 2 | 0 | 1 | 0 | 1 | 1 | 2 | 0 | 2 | 2 | 10 | 3 | 310119 |
| 120 | 0 | 0 | 2 | 1 | 2 | 1 | 1 | 0 | 0 | 0 | 10 | 3 | 310120 |
| 1 | 2 | 2 | 1 | 0 | 3 | 0 | 0 | 0 | 3 | 2 | 11 | 2 | 201001 |
| 2 | 1 | 0 | 2 | 2 | 0 | 2 | 2 | 0 | 0 | 1 | 11 | 2 | 201002 |
| 3 | 0 | 0 | 0 | 3 | 1 | 2 | 1 | 1 | 1 | 3 | 11 | 2 | 201003 |
| 4 | 1 | 0 | 1 | 0 | 3 | 2 | 2 | 2 | 2 | 2 | 11 | 2 | 201004 |
| 5 | 0 | 1 | 0 | 1 | 3 | 1 | 2 | 1 | 2 | 1 | 11 | 3 | 301005 |
| 6 | 1 | 1 | 2 | 3 | 2 | 2 | 0 | 0 | 2 | 1 | 11 | 3 | 301006 |
| 7 | 2 | 1 | 0 | 2 | 3 | 1 | 2 | 1 | 3 | 0 | 11 | 3 | 301007 |
| 8 | 1 | 2 | 1 | 0 | 3 | 2 | 1 | 2 | 1 | 0 | 11 | 3 | 301008 |
| 9 | 1 | 1 | 2 | 3 | 0 | 0 | 2 | 0 | 1 | 2 | 11 | 1 | 101009 |
| 10 | 2 | 0 | 0 | 3 | 1 | 1 | 2 | 1 | 1 | 3 | 11 | 1 | 101010 |
| 11 | 0 | 1 | 1 | 1 | 1 | 2 | 0 | 2 | 0 | 0 | 11 | 1 | 101011 |
| 12 | 1 | 2 | 2 | 1 | 3 | 0 | 1 | 1 | 2 | 2 | 11 | 1 | 101012 |
| 13 | 1 | 2 | 0 | 1 | 2 | 2 | 1 | 1 | 2 | 0 | 12 | 2 | 202013 |
| 14 | 1 | 1 | 1 | 0 | 0 | 0 | 0 | 2 | 1 | 3 | 12 | 2 | 202014 |
| 15 | 1 | 1 | 2 | 2 | 1 | 0 | 2 | 1 | 0 | 3 | 12 | 2 | 202015 |
| 16 | 1 | 2 | 2 | 2 | 0 | 2 | 0 | 1 | 0 | 2 | 12 | 2 | 202016 |
| 17 | 1 | 0 | 1 | 1 | 0 | 0 | 2 | 2 | 3 | 3 | 12 | 3 | 302017 |
| 18 | 0 | 1 | 2 | 1 | 0 | 2 | 0 | 0 | 2 | 3 | 12 | 3 | 302018 |
| 19 | 0 | 0 | 2 | 0 | 2 | 2 | 1 | 0 | 1 | 0 | 12 | 3 | 302019 |
| 20 | 2 | 1 | 1 | 2 | 2 | 1 | 0 | 2 | 3 | 0 | 12 | 3 | 302020 |
| 21 | 1 | 1 | 0 | 2 | 2 | 0 | 0 | 2 | 1 | 1 | 12 | 1 | 102021 |
| 22 | 0 | 0 | 1 | 3 | 0 | 1 | 1 | 2 | 0 | 1 | 12 | 1 | 102022 |
| 23 | 2 | 0 | 2 | 2 | 0 | 0 | 2 | 1 | 1 | 1 | 12 | 1 | 102023 |
| 24 | 1 | 1 | 0 | 0 | 2 | 2 | 0 | 1 | 1 | 0 | 12 | 1 | 102024 |
| 25 | 2 | 1 | 0 | 3 | 2 | 0 | 2 | 2 | 2 | 3 | 13 | 2 | 203025 |
| 26 | 2 | 1 | 2 | 0 | 0 | 0 | 0 | 1 | 3 | 1 | 13 | 2 | 203026 |
| 27 | 2 | 0 | 1 | 2 | 2 | 1 | 2 | 0 | 0 | 3 | 13 | 2 | 203027 |
| 28 | 0 | 2 | 2 | 0 | 3 | 2 | 0 | 0 | 3 | 0 | 13 | 2 | 203028 |
| 29 | 2 | 2 | 0 | 0 | 2 | 0 | 1 | 2 | 2 | 0 | 13 | 3 | 303029 |
| 30 | 2 | 2 | 2 | 1 | 0 | 0 | 1 | 1 | 0 | 2 | 13 | 3 | 303030 |
| 31 | 0 | 2 | 0 | 2 | 0 | 2 | 0 | 2 | 3 | 3 | 13 | 3 | 303031 |
| 32 | 2 | 2 | 1 | 1 | 3 | 1 | 1 | 0 | 2 | 1 | 13 | 3 | 303032 |
| 33 | 2 | 2 | 2 | 3 | 2 | 1 | 1 | 1 | 0 | 1 | 13 | 1 | 103033 |
| 34 | 0 | 0 | 0 | 2 | 0 | 1 | 2 | 1 | 3 | 1 | 13 | 1 | 103034 |
| 35 | 0 | 0 | 0 | 0 | 3 | 2 | 1 | 1 | 2 | 1 | 13 | 1 | 103035 |
| 36 | 2 | 2 | 0 | 2 | 3 | 0 | 1 | 1 | 3 | 2 | 13 | 1 | 103036 |
| 37 | 1 | 0 | 1 | 2 | 2 | 0 | 1 | 0 | 0 | 0 | 14 | 2 | 204037 |
| 38 | 2 | 0 | 1 | 0 | 2 | 0 | 2 | 0 | 3 | 0 | 14 | 2 | 204038 |
| 39 | 1 | 2 | 0 | 3 | 2 | 0 | 0 | 2 | 1 | 0 | 14 | 2 | 204039 |
| 40 | 0 | 2 | 0 | 3 | 0 | 2 | 1 | 2 | 0 | 3 | 14 | 2 | 204040 |
| 41 | 0 | 0 | 2 | 0 | 1 | 2 | 1 | 1 | 3 | 0 | 14 | 3 | 304041 |
| 42 | 0 | 0 | 2 | 0 | 2 | 1 | 2 | 0 | 2 | 3 | 14 | 3 | 304042 |
| 43 | 1 | 2 | 2 | 1 | 1 | 0 | 0 | 1 | 3 | 3 | 14 | 3 | 304043 |
| 44 | 0 | 2 | 1 | 2 | 1 | 2 | 0 | 2 | 0 | 3 | 14 | 3 | 304044 |
| 45 | 0 | 2 | 2 | 2 | 2 | 2 | 0 | 0 | 1 | 0 | 14 | 1 | 104045 |
| 46 | 0 | 0 | 1 | 3 | 2 | 1 | 1 | 0 | 2 | 1 | 14 | 1 | 104046 |
| 47 | 2 | 2 | 0 | 1 | 2 | 1 | 0 | 2 | 3 | 3 | 14 | 1 | 104047 |
| 48 | 0 | 1 | 0 | 2 | 2 | 2 | 2 | 2 | 1 | 1 | 14 | 1 | 104048 |
| 49 | 1 | 1 | 2 | 3 | 2 | 0 | 0 | 1 | 2 | 1 | 15 | 2 | 205049 |
| 50 | 1 | 1 | 2 | 2 | 1 | 2 | 2 | 0 | 1 | 2 | 15 | 2 | 205050 |
| 51 | 0 | 1 | 1 | 1 | 2 | 1 | 2 | 0 | 0 | 1 | 15 | 2 | 205051 |
| 52 | 0 | 1 | 2 | 3 | 3 | 1 | 0 | 0 | 2 | 1 | 15 | 2 | 205052 |
| 53 | 2 | 2 | 1 | 0 | 0 | 1 | 0 | 0 | 1 | 1 | 15 | 3 | 305053 |
| 54 | 0 | 0 | 1 | 3 | 1 | 1 | 2 | 0 | 1 | 2 | 15 | 3 | 305054 |
| 55 | 2 | 2 | 1 | 2 | 1 | 0 | 1 | 2 | 0 | 0 | 15 | 3 | 305055 |
| 56 | 0 | 0 | 2 | 1 | 3 | 1 | 1 | 0 | 0 | 0 | 15 | 3 | 305056 |
| 57 | 1 | 2 | 1 | 3 | 0 | 0 | 1 | 2 | 1 | 1 | 15 | 1 | 105057 |
| 58 | 0 | 1 | 0 | 0 | 0 | 1 | 0 | 2 | 3 | 2 | 15 | 1 | 105058 |
| 59 | 2 | 0 | 0 | 3 | 0 | 1 | 1 | 1 | 1 | 2 | 15 | 1 | 105059 |
| 60 | 1 | 0 | 2 | 2 | 3 | 2 | 2 | 0 | 0 | 0 | 15 | 1 | 105060 |
| 61 | 0 | 1 | 1 | 0 | 3 | 2 | 0 | 2 | 2 | 2 | 16 | 2 | 206061 |
| 62 | 2 | 2 | 2 | 3 | 0 | 0 | 1 | 0 | 0 | 1 | 16 | 2 | 206062 |
| 63 | 1 | 1 | 1 | 1 | 2 | 2 | 2 | 2 | 3 | 1 | 16 | 2 | 206063 |
| 64 | 2 | 2 | 2 | 0 | 1 | 1 | 0 | 0 | 2 | 3 | 16 | 2 | 206064 |
| 65 | 0 | 0 | 0 | 1 | 1 | 1 | 2 | 2 | 0 | 2 | 16 | 3 | 306065 |
| 66 | 0 | 0 | 0 | 0 | 2 | 1 | 1 | 2 | 1 | 3 | 16 | 3 | 306066 |
| 67 | 2 | 2 | 1 | 2 | 0 | 0 | 0 | 0 | 0 | 1 | 16 | 3 | 306067 |
| 68 | 1 | 0 | 2 | 3 | 1 | 2 | 2 | 0 | 1 | 2 | 16 | 3 | 306068 |
| 69 | 2 | 2 | 1 | 2 | 3 | 1 | 1 | 2 | 3 | 2 | 16 | 1 | 106069 |
| 70 | 0 | 2 | 1 | 1 | 0 | 1 | 0 | 0 | 3 | 1 | 16 | 1 | 106070 |
| 71 | 1 | 0 | 2 | 2 | 2 | 2 | 1 | 1 | 3 | 1 | 16 | 1 | 106071 |
| 72 | 1 | 0 | 0 | 3 | 3 | 0 | 2 | 2 | 0 | 0 | 16 | 1 | 106072 |
| 73 | 2 | 1 | 0 | 0 | 1 | 0 | 2 | 1 | 3 | 3 | 17 | 2 | 207073 |
| 74 | 1 | 1 | 0 | 1 | 1 | 0 | 2 | 2 | 2 | 0 | 17 | 2 | 207074 |
| 75 | 2 | 0 | 0 | 1 | 2 | 0 | 2 | 1 | 2 | 3 | 17 | 2 | 207075 |
| 76 | 2 | 0 | 0 | 3 | 0 | 1 | 2 | 1 | 1 | 3 | 17 | 2 | 207076 |
| 77 | 2 | 1 | 1 | 0 | 1 | 1 | 2 | 0 | 1 | 2 | 17 | 3 | 307077 |
| 78 | 1 | 1 | 1 | 1 | 3 | 0 | 2 | 2 | 2 | 1 | 17 | 3 | 307078 |
| 79 | 2 | 1 | 1 | 2 | 1 | 1 | 0 | 0 | 1 | 0 | 17 | 3 | 307079 |
| 80 | 0 | 2 | 2 | 1 | 0 | 2 | 0 | 1 | 2 | 2 | 17 | 3 | 307080 |
| 81 | 1 | 0 | 1 | 1 | 1 | 0 | 1 | 0 | 0 | 3 | 17 | 1 | 107081 |
| 82 | 2 | 0 | 2 | 1 | 1 | 1 | 1 | 1 | 3 | 3 | 17 | 1 | 107082 |
| 83 | 0 | 2 | 0 | 3 | 0 | 1 | 0 | 1 | 1 | 3 | 17 | 1 | 107083 |
| 84 | 2 | 1 | 0 | 3 | 3 | 1 | 0 | 1 | 1 | 0 | 17 | 1 | 107084 |
| 85 | 0 | 1 | 0 | 3 | 1 | 1 | 0 | 2 | 0 | 3 | 18 | 2 | 208085 |
| 86 | 1 | 2 | 0 | 1 | 1 | 2 | 1 | 1 | 2 | 3 | 18 | 2 | 208086 |
| 87 | 2 | 1 | 2 | 3 | 1 | 0 | 0 | 0 | 0 | 2 | 18 | 2 | 208087 |
| 88 | 1 | 1 | 2 | 3 | 3 | 0 | 0 | 1 | 2 | 0 | 18 | 2 | 208088 |
| 89 | 2 | 2 | 1 | 0 | 2 | 0 | 0 | 0 | 2 | 3 | 18 | 3 | 308089 |
| 90 | 1 | 2 | 0 | 0 | 1 | 0 | 0 | 1 | 1 | 2 | 18 | 3 | 308090 |
| 91 | 2 | 0 | 0 | 0 | 3 | 0 | 1 | 1 | 2 | 2 | 18 | 3 | 308091 |
| 92 | 1 | 0 | 0 | 2 | 3 | 0 | 1 | 2 | 3 | 1 | 18 | 3 | 308092 |
| 93 | 1 | 0 | 2 | 0 | 0 | 2 | 1 | 1 | 3 | 3 | 18 | 1 | 108093 |
| 94 | 2 | 0 | 2 | 0 | 3 | 0 | 2 | 0 | 2 | 1 | 18 | 1 | 108094 |
| 95 | 1 | 1 | 2 | 2 | 2 | 2 | 2 | 0 | 3 | 3 | 18 | 1 | 108095 |
| 96 | 0 | 1 | 0 | 1 | 3 | 1 | 2 | 2 | 0 | 2 | 18 | 1 | 108096 |
| 97 | 1 | 0 | 0 | 0 | 0 | 2 | 1 | 2 | 3 | 1 | 19 | 2 | 209097 |
| 98 | 1 | 2 | 1 | 3 | 1 | 2 | 1 | 0 | 1 | 2 | 19 | 2 | 209098 |
| 99 | 1 | 0 | 1 | 3 | 0 | 2 | 1 | 2 | 0 | 1 | 19 | 2 | 209099 |
| 100 | 2 | 1 | 2 | 3 | 2 | 0 | 0 | 1 | 0 | 1 | 19 | 2 | 209100 |
| 101 | 0 | 1 | 2 | 1 | 0 | 1 | 2 | 1 | 3 | 2 | 19 | 3 | 309101 |
| 102 | 1 | 2 | 1 | 1 | 1 | 0 | 1 | 0 | 3 | 2 | 19 | 3 | 309102 |
| 103 | 0 | 1 | 0 | 2 | 2 | 2 | 2 | 1 | 3 | 0 | 19 | 3 | 309103 |
| 104 | 0 | 2 | 0 | 2 | 3 | 1 | 0 | 1 | 3 | 2 | 19 | 3 | 309104 |
| 105 | 2 | 2 | 2 | 0 | 2 | 1 | 1 | 0 | 3 | 0 | 19 | 1 | 109105 |
| 106 | 2 | 2 | 0 | 2 | 3 | 1 | 1 | 2 | 0 | 0 | 19 | 1 | 109106 |
| 107 | 2 | 2 | 1 | 0 | 0 | 1 | 1 | 2 | 1 | 3 | 19 | 1 | 109107 |
| 108 | 0 | 0 | 2 | 0 | 1 | 1 | 2 | 0 | 1 | 0 | 19 | 1 | 109108 |
| 109 | 1 | 0 | 1 | 2 | 3 | 0 | 2 | 2 | 1 | 2 | 20 | 2 | 210109 |
| 110 | 0 | 1 | 1 | 3 | 3 | 2 | 0 | 2 | 2 | 2 | 20 | 2 | 210110 |
| 111 | 2 | 1 | 0 | 1 | 3 | 0 | 2 | 1 | 0 | 2 | 20 | 2 | 210111 |
| 112 | 1 | 1 | 1 | 1 | 0 | 2 | 2 | 2 | 2 | 3 | 20 | 2 | 210112 |
| 113 | 0 | 2 | 2 | 2 | 3 | 2 | 0 | 1 | 1 | 1 | 20 | 3 | 310113 |
| 114 | 1 | 2 | 1 | 1 | 1 | 2 | 1 | 2 | 2 | 3 | 20 | 3 | 310114 |
| 115 | 2 | 1 | 1 | 1 | 1 | 0 | 0 | 2 | 0 | 0 | 20 | 3 | 310115 |
| 116 | 0 | 0 | 0 | 3 | 3 | 2 | 1 | 2 | 2 | 0 | 20 | 3 | 310116 |
| 117 | 0 | 2 | 2 | 3 | 1 | 2 | 0 | 1 | 0 | 0 | 20 | 1 | 110117 |
| 118 | 0 | 1 | 1 | 2 | 0 | 2 | 2 | 0 | 3 | 2 | 20 | 1 | 110118 |
| 119 | 2 | 0 | 1 | 0 | 1 | 1 | 2 | 0 | 2 | 2 | 20 | 1 | 110119 |
| 120 | 0 | 0 | 2 | 1 | 2 | 1 | 1 | 0 | 0 | 0 | 20 | 1 | 110120 |
| 1 | 2 | 2 | 1 | 0 | 3 | 0 | 0 | 0 | 3 | 2 | 21 | 3 | 301001 |
| 2 | 1 | 0 | 2 | 2 | 0 | 2 | 2 | 0 | 0 | 1 | 21 | 3 | 301002 |
| 3 | 0 | 0 | 0 | 3 | 1 | 2 | 1 | 1 | 1 | 3 | 21 | 3 | 301003 |
| 4 | 1 | 0 | 1 | 0 | 3 | 2 | 2 | 2 | 2 | 2 | 21 | 3 | 301004 |
| 5 | 0 | 1 | 0 | 1 | 3 | 1 | 2 | 1 | 2 | 1 | 21 | 1 | 101005 |
| 6 | 1 | 1 | 2 | 3 | 2 | 2 | 0 | 0 | 2 | 1 | 21 | 1 | 101006 |
| 7 | 2 | 1 | 0 | 2 | 3 | 1 | 2 | 1 | 3 | 0 | 21 | 1 | 101007 |
| 8 | 1 | 2 | 1 | 0 | 3 | 2 | 1 | 2 | 1 | 0 | 21 | 1 | 101008 |
| 9 | 1 | 1 | 2 | 3 | 0 | 0 | 2 | 0 | 1 | 2 | 21 | 2 | 201009 |
| 10 | 2 | 0 | 0 | 3 | 1 | 1 | 2 | 1 | 1 | 3 | 21 | 2 | 201010 |
| 11 | 0 | 1 | 1 | 1 | 1 | 2 | 0 | 2 | 0 | 0 | 21 | 2 | 201011 |
| 12 | 1 | 2 | 2 | 1 | 3 | 0 | 1 | 1 | 2 | 2 | 21 | 2 | 201012 |
| 13 | 1 | 2 | 0 | 1 | 2 | 2 | 1 | 1 | 2 | 0 | 22 | 3 | 302013 |
| 14 | 1 | 1 | 1 | 0 | 0 | 0 | 0 | 2 | 1 | 3 | 22 | 3 | 302014 |
| 15 | 1 | 1 | 2 | 2 | 1 | 0 | 2 | 1 | 0 | 3 | 22 | 3 | 302015 |
| 16 | 1 | 2 | 2 | 2 | 0 | 2 | 0 | 1 | 0 | 2 | 22 | 3 | 302016 |
| 17 | 1 | 0 | 1 | 1 | 0 | 0 | 2 | 2 | 3 | 3 | 22 | 1 | 102017 |
| 18 | 0 | 1 | 2 | 1 | 0 | 2 | 0 | 0 | 2 | 3 | 22 | 1 | 102018 |
| 19 | 0 | 0 | 2 | 0 | 2 | 2 | 1 | 0 | 1 | 0 | 22 | 1 | 102019 |
| 20 | 2 | 1 | 1 | 2 | 2 | 1 | 0 | 2 | 3 | 0 | 22 | 1 | 102020 |
| 21 | 1 | 1 | 0 | 2 | 2 | 0 | 0 | 2 | 1 | 1 | 22 | 2 | 202021 |
| 22 | 0 | 0 | 1 | 3 | 0 | 1 | 1 | 2 | 0 | 1 | 22 | 2 | 202022 |
| 23 | 2 | 0 | 2 | 2 | 0 | 0 | 2 | 1 | 1 | 1 | 22 | 2 | 202023 |
| 24 | 1 | 1 | 0 | 0 | 2 | 2 | 0 | 1 | 1 | 0 | 22 | 2 | 202024 |
| 25 | 2 | 1 | 0 | 3 | 2 | 0 | 2 | 2 | 2 | 3 | 23 | 3 | 303025 |
| 26 | 2 | 1 | 2 | 0 | 0 | 0 | 0 | 1 | 3 | 1 | 23 | 3 | 303026 |
| 27 | 2 | 0 | 1 | 2 | 2 | 1 | 2 | 0 | 0 | 3 | 23 | 3 | 303027 |
| 28 | 0 | 2 | 2 | 0 | 3 | 2 | 0 | 0 | 3 | 0 | 23 | 3 | 303028 |
| 29 | 2 | 2 | 0 | 0 | 2 | 0 | 1 | 2 | 2 | 0 | 23 | 1 | 103029 |
| 30 | 2 | 2 | 2 | 1 | 0 | 0 | 1 | 1 | 0 | 2 | 23 | 1 | 103030 |
| 31 | 0 | 2 | 0 | 2 | 0 | 2 | 0 | 2 | 3 | 3 | 23 | 1 | 103031 |
| 32 | 2 | 2 | 1 | 1 | 3 | 1 | 1 | 0 | 2 | 1 | 23 | 1 | 103032 |
| 33 | 2 | 2 | 2 | 3 | 2 | 1 | 1 | 1 | 0 | 1 | 23 | 2 | 203033 |
| 34 | 0 | 0 | 0 | 2 | 0 | 1 | 2 | 1 | 3 | 1 | 23 | 2 | 203034 |
| 35 | 0 | 0 | 0 | 0 | 3 | 2 | 1 | 1 | 2 | 1 | 23 | 2 | 203035 |
| 36 | 2 | 2 | 0 | 2 | 3 | 0 | 1 | 1 | 3 | 2 | 23 | 2 | 203036 |
| 37 | 1 | 0 | 1 | 2 | 2 | 0 | 1 | 0 | 0 | 0 | 24 | 3 | 304037 |
| 38 | 2 | 0 | 1 | 0 | 2 | 0 | 2 | 0 | 3 | 0 | 24 | 3 | 304038 |
| 39 | 1 | 2 | 0 | 3 | 2 | 0 | 0 | 2 | 1 | 0 | 24 | 3 | 304039 |
| 40 | 0 | 2 | 0 | 3 | 0 | 2 | 1 | 2 | 0 | 3 | 24 | 3 | 304040 |
| 41 | 0 | 0 | 2 | 0 | 1 | 2 | 1 | 1 | 3 | 0 | 24 | 1 | 104041 |
| 42 | 0 | 0 | 2 | 0 | 2 | 1 | 2 | 0 | 2 | 3 | 24 | 1 | 104042 |
| 43 | 1 | 2 | 2 | 1 | 1 | 0 | 0 | 1 | 3 | 3 | 24 | 1 | 104043 |
| 44 | 0 | 2 | 1 | 2 | 1 | 2 | 0 | 2 | 0 | 3 | 24 | 1 | 104044 |
| 45 | 0 | 2 | 2 | 2 | 2 | 2 | 0 | 0 | 1 | 0 | 24 | 2 | 204045 |
| 46 | 0 | 0 | 1 | 3 | 2 | 1 | 1 | 0 | 2 | 1 | 24 | 2 | 204046 |
| 47 | 2 | 2 | 0 | 1 | 2 | 1 | 0 | 2 | 3 | 3 | 24 | 2 | 204047 |
| 48 | 0 | 1 | 0 | 2 | 2 | 2 | 2 | 2 | 1 | 1 | 24 | 2 | 204048 |
| 49 | 1 | 1 | 2 | 3 | 2 | 0 | 0 | 1 | 2 | 1 | 25 | 3 | 305049 |
| 50 | 1 | 1 | 2 | 2 | 1 | 2 | 2 | 0 | 1 | 2 | 25 | 3 | 305050 |
| 51 | 0 | 1 | 1 | 1 | 2 | 1 | 2 | 0 | 0 | 1 | 25 | 3 | 305051 |
| 52 | 0 | 1 | 2 | 3 | 3 | 1 | 0 | 0 | 2 | 1 | 25 | 3 | 305052 |
| 53 | 2 | 2 | 1 | 0 | 0 | 1 | 0 | 0 | 1 | 1 | 25 | 1 | 105053 |
| 54 | 0 | 0 | 1 | 3 | 1 | 1 | 2 | 0 | 1 | 2 | 25 | 1 | 105054 |
| 55 | 2 | 2 | 1 | 2 | 1 | 0 | 1 | 2 | 0 | 0 | 25 | 1 | 105055 |
| 56 | 0 | 0 | 2 | 1 | 3 | 1 | 1 | 0 | 0 | 0 | 25 | 1 | 105056 |
| 57 | 1 | 2 | 1 | 3 | 0 | 0 | 1 | 2 | 1 | 1 | 25 | 2 | 205057 |
| 58 | 0 | 1 | 0 | 0 | 0 | 1 | 0 | 2 | 3 | 2 | 25 | 2 | 205058 |
| 59 | 2 | 0 | 0 | 3 | 0 | 1 | 1 | 1 | 1 | 2 | 25 | 2 | 205059 |
| 60 | 1 | 0 | 2 | 2 | 3 | 2 | 2 | 0 | 0 | 0 | 25 | 2 | 205060 |
| 61 | 0 | 1 | 1 | 0 | 3 | 2 | 0 | 2 | 2 | 2 | 26 | 3 | 306061 |
| 62 | 2 | 2 | 2 | 3 | 0 | 0 | 1 | 0 | 0 | 1 | 26 | 3 | 306062 |
| 63 | 1 | 1 | 1 | 1 | 2 | 2 | 2 | 2 | 3 | 1 | 26 | 3 | 306063 |
| 64 | 2 | 2 | 2 | 0 | 1 | 1 | 0 | 0 | 2 | 3 | 26 | 3 | 306064 |
| 65 | 0 | 0 | 0 | 1 | 1 | 1 | 2 | 2 | 0 | 2 | 26 | 1 | 106065 |
| 66 | 0 | 0 | 0 | 0 | 2 | 1 | 1 | 2 | 1 | 3 | 26 | 1 | 106066 |
| 67 | 2 | 2 | 1 | 2 | 0 | 0 | 0 | 0 | 0 | 1 | 26 | 1 | 106067 |
| 68 | 1 | 0 | 2 | 3 | 1 | 2 | 2 | 0 | 1 | 2 | 26 | 1 | 106068 |
| 69 | 2 | 2 | 1 | 2 | 3 | 1 | 1 | 2 | 3 | 2 | 26 | 2 | 206069 |
| 70 | 0 | 2 | 1 | 1 | 0 | 1 | 0 | 0 | 3 | 1 | 26 | 2 | 206070 |
| 71 | 1 | 0 | 2 | 2 | 2 | 2 | 1 | 1 | 3 | 1 | 26 | 2 | 206071 |
| 72 | 1 | 0 | 0 | 3 | 3 | 0 | 2 | 2 | 0 | 0 | 26 | 2 | 206072 |
| 73 | 2 | 1 | 0 | 0 | 1 | 0 | 2 | 1 | 3 | 3 | 27 | 3 | 307073 |
| 74 | 1 | 1 | 0 | 1 | 1 | 0 | 2 | 2 | 2 | 0 | 27 | 3 | 307074 |
| 75 | 2 | 0 | 0 | 1 | 2 | 0 | 2 | 1 | 2 | 3 | 27 | 3 | 307075 |
| 76 | 2 | 0 | 0 | 3 | 0 | 1 | 2 | 1 | 1 | 3 | 27 | 3 | 307076 |
| 77 | 2 | 1 | 1 | 0 | 1 | 1 | 2 | 0 | 1 | 2 | 27 | 1 | 107077 |
| 78 | 1 | 1 | 1 | 1 | 3 | 0 | 2 | 2 | 2 | 1 | 27 | 1 | 107078 |
| 79 | 2 | 1 | 1 | 2 | 1 | 1 | 0 | 0 | 1 | 0 | 27 | 1 | 107079 |
| 80 | 0 | 2 | 2 | 1 | 0 | 2 | 0 | 1 | 2 | 2 | 27 | 1 | 107080 |
| 81 | 1 | 0 | 1 | 1 | 1 | 0 | 1 | 0 | 0 | 3 | 27 | 2 | 207081 |
| 82 | 2 | 0 | 2 | 1 | 1 | 1 | 1 | 1 | 3 | 3 | 27 | 2 | 207082 |
| 83 | 0 | 2 | 0 | 3 | 0 | 1 | 0 | 1 | 1 | 3 | 27 | 2 | 207083 |
| 84 | 2 | 1 | 0 | 3 | 3 | 1 | 0 | 1 | 1 | 0 | 27 | 2 | 207084 |
| 85 | 0 | 1 | 0 | 3 | 1 | 1 | 0 | 2 | 0 | 3 | 28 | 3 | 308085 |
| 86 | 1 | 2 | 0 | 1 | 1 | 2 | 1 | 1 | 2 | 3 | 28 | 3 | 308086 |
| 87 | 2 | 1 | 2 | 3 | 1 | 0 | 0 | 0 | 0 | 2 | 28 | 3 | 308087 |
| 88 | 1 | 1 | 2 | 3 | 3 | 0 | 0 | 1 | 2 | 0 | 28 | 3 | 308088 |
| 89 | 2 | 2 | 1 | 0 | 2 | 0 | 0 | 0 | 2 | 3 | 28 | 1 | 108089 |
| 90 | 1 | 2 | 0 | 0 | 1 | 0 | 0 | 1 | 1 | 2 | 28 | 1 | 108090 |
| 91 | 2 | 0 | 0 | 0 | 3 | 0 | 1 | 1 | 2 | 2 | 28 | 1 | 108091 |
| 92 | 1 | 0 | 0 | 2 | 3 | 0 | 1 | 2 | 3 | 1 | 28 | 1 | 108092 |
| 93 | 1 | 0 | 2 | 0 | 0 | 2 | 1 | 1 | 3 | 3 | 28 | 2 | 208093 |
| 94 | 2 | 0 | 2 | 0 | 3 | 0 | 2 | 0 | 2 | 1 | 28 | 2 | 208094 |
| 95 | 1 | 1 | 2 | 2 | 2 | 2 | 2 | 0 | 3 | 3 | 28 | 2 | 208095 |
| 96 | 0 | 1 | 0 | 1 | 3 | 1 | 2 | 2 | 0 | 2 | 28 | 2 | 208096 |
| 97 | 1 | 0 | 0 | 0 | 0 | 2 | 1 | 2 | 3 | 1 | 29 | 3 | 309097 |
| 98 | 1 | 2 | 1 | 3 | 1 | 2 | 1 | 0 | 1 | 2 | 29 | 3 | 309098 |
| 99 | 1 | 0 | 1 | 3 | 0 | 2 | 1 | 2 | 0 | 1 | 29 | 3 | 309099 |
| 100 | 2 | 1 | 2 | 3 | 2 | 0 | 0 | 1 | 0 | 1 | 29 | 3 | 309100 |
| 101 | 0 | 1 | 2 | 1 | 0 | 1 | 2 | 1 | 3 | 2 | 29 | 1 | 109101 |
| 102 | 1 | 2 | 1 | 1 | 1 | 0 | 1 | 0 | 3 | 2 | 29 | 1 | 109102 |
| 103 | 0 | 1 | 0 | 2 | 2 | 2 | 2 | 1 | 3 | 0 | 29 | 1 | 109103 |
| 104 | 0 | 2 | 0 | 2 | 3 | 1 | 0 | 1 | 3 | 2 | 29 | 1 | 109104 |
| 105 | 2 | 2 | 2 | 0 | 2 | 1 | 1 | 0 | 3 | 0 | 29 | 2 | 209105 |
| 106 | 2 | 2 | 0 | 2 | 3 | 1 | 1 | 2 | 0 | 0 | 29 | 2 | 209106 |
| 107 | 2 | 2 | 1 | 0 | 0 | 1 | 1 | 2 | 1 | 3 | 29 | 2 | 209107 |
| 108 | 0 | 0 | 2 | 0 | 1 | 1 | 2 | 0 | 1 | 0 | 29 | 2 | 209108 |
| 109 | 1 | 0 | 1 | 2 | 3 | 0 | 2 | 2 | 1 | 2 | 30 | 3 | 310109 |
| 110 | 0 | 1 | 1 | 3 | 3 | 2 | 0 | 2 | 2 | 2 | 30 | 3 | 310110 |
| 111 | 2 | 1 | 0 | 1 | 3 | 0 | 2 | 1 | 0 | 2 | 30 | 3 | 310111 |
| 112 | 1 | 1 | 1 | 1 | 0 | 2 | 2 | 2 | 2 | 3 | 30 | 3 | 310112 |
| 113 | 0 | 2 | 2 | 2 | 3 | 2 | 0 | 1 | 1 | 1 | 30 | 1 | 110113 |
| 114 | 1 | 2 | 1 | 1 | 1 | 2 | 1 | 2 | 2 | 3 | 30 | 1 | 110114 |
| 115 | 2 | 1 | 1 | 1 | 1 | 0 | 0 | 2 | 0 | 0 | 30 | 1 | 110115 |
| 116 | 0 | 0 | 0 | 3 | 3 | 2 | 1 | 2 | 2 | 0 | 30 | 1 | 110116 |
| 117 | 0 | 2 | 2 | 3 | 1 | 2 | 0 | 1 | 0 | 0 | 30 | 2 | 210117 |
| 118 | 0 | 1 | 1 | 2 | 0 | 2 | 2 | 0 | 3 | 2 | 30 | 2 | 210118 |
| 119 | 2 | 0 | 1 | 0 | 1 | 1 | 2 | 0 | 2 | 2 | 30 | 2 | 210119 |
| 120 | 0 | 0 | 2 | 1 | 2 | 1 | 1 | 0 | 0 | 0 | 30 | 2 | 210120 |
