# Supplementary material for: Patient preferences for Remote cochlear implant management: A discrete choice experiment
Source: PLoS One. 2025 Jun 3;20(6):e0320421. doi: 10.1371/journal.pone.0320421 (PMC12133006; doi:10.1371/journal.pone.0320421)
Supplement: S4 Table — Participants with ≥95% survey progress were included in data analysis. (DOCX) [file pone.0320421.s005.docx]

Table S4: DCE block allocation for all participants who started the DCE survey. Participants with ≥95% survey progress were included in data analysis. Surveys were initiated between 21/07/2022 and 3/12/2022

| Survey Progress | Time (in seconds) to complete survey | Finished survey? | Initial Participant ID | DCE Block allocated |
| --- | --- | --- | --- | --- |
| 100 | 2967 | True | DCEI001 | 22 |
| 100 | 22627 | True | DCEI002 | 1 |
| 100 | 1130 | True | DCEI003 | 18 |
| 100 | 2029 | True | DCEI004 | 20 |
| 100 | 819 | True | DCEI005 | 10 |
| 100 | 929 | True | DCEI006 | 30 |
| 100 | 877 | True | DCEI007 | 5 |
| 100 | 1216 | True | DCEI008 | 25 |
| 100 | 1120 | True | DCEI009 | 23 |
| 100 | 992 | True | DCEI010 | 19 |
| 100 | 755 | True | DCEI011 | 9 |
| 100 | 422 | True | DCEI012 | 6 |
| 100 | 2680 | True | DCEI013 | 29 |
| 100 | 1070 | True | DCEI014 | 12 |
| 100 | 475 | True | DCEI015 | 17 |
| 100 | 1323 | True | DCEI016 | 14 |
| 100 | 1633 | True | DCEI017 | 13 |
| 100 | 1930 | True | DCEI018 | 19 |
| 100 | 24778 | True | DCEI019 | 26 |
| 100 | 1381 | True | DCEI020 | 6 |
| 100 | 1345 | True | DCEI021 | 20 |
| 100 | 2082 | True | DCEI022 | 7 |
| 100 | 1025 | True | DCEI023 | 30 |
| 100 | 2294 | True | DCEI024 | 24 |
| 100 | 1223 | True | DCEI025 | 8 |
| 100 | 2293 | True | DCEI026 | 22 |
| 100 | 3188 | True | DCEI027 | 10 |
| 100 | 633 | True | DCEI028 | 12 |
| 100 | 973 | True | DCEI029 | 21 |
| 100 | 21657 | True | DCEI030 | 2 |
| 100 | 4720 | True | DCEI031 | 17 |
| 100 | 499484 | True | DCEI032 | 27 |
| 100 | 2084 | True | DCEI033 | 15 |
| 100 | 543932 | True | DCEI034 | 16 |
| 100 | 1797 | True | DCEI035 | 11 |
| 100 | 1216 | True | DCEI036 | 23 |
| 100 | 1356 | True | DCEI037 | 26 |
| 100 | 987 | True | DCEI038 | 28 |
| 100 | 1596 | True | DCEI039 | 3 |
| 100 | 990 | True | DCEI040 | 1 |
| 100 | 3098 | True | DCEI041 | 3 |
| 100 | 789 | True | DCEI042 | 17 |
| 100 | 4420 | True | DCEI043 | 29 |
| 100 | 1162014 | True | DCEI044 | 25 |
| 100 | 1607 | True | DCEI045 | 1 |
| 100 | 776 | True | DCEI046 | 6 |
| 100 | 856 | True | DCEI047 | 2 |
| 100 | 1180 | True | DCEI048 | 21 |
| 100 | 750 | True | DCEI049 | 8 |
| 100 | 19930 | True | DCEI050 | 4 |
| 100 | 33990 | True | DCEI051 | 10 |
| 100 | 1690 | True | DCEI052 | 30 |
| 100 | 5743 | True | DCEI053 | 16 |
| 100 | 8797 | True | DCEI054 | 7 |
| 100 | 3109 | True | DCEI055 | 26 |
| 100 | 2117 | True | DCEI056 | 27 |
| 100 | 76693 | True | DCEI057 | 5 |
| 100 | 665 | True | DCEI058 | 19 |
| 100 | 646 | True | DCEI059 | 11 |
| 100 | 1190 | True | DCEI060 | 25 |
| 100 | 902 | True | DCEI061 | 12 |
| 100 | 981 | True | DCEI062 | 24 |
| 100 | 751 | True | DCEI063 | 15 |
| 100 | 1174 | True | DCEI064 | 28 |
| 100 | 499 | True | DCEI065 | 10 |
| 100 | 482 | True | DCEI066 | 6 |
| 100 | 1333 | True | DCEI067 | 23 |
| 100 | 1685 | True | DCEI068 | 22 |
| 100 | 2596 | True | DCEI069 | 9 |
| 100 | 1279 | True | DCEI070 | 24 |
| 100 | 1353 | True | DCEI071 | 15 |
| 100 | 2567 | True | DCEI072 | 25 |
| 100 | 1276 | True | DCEI073 | 3 |
| 100 | 4792 | True | DCEI074 | 12 |
| 100 | 1031 | True | DCEI075 | 16 |
| 100 | 1657 | True | DCEI076 | 4 |
| 100 | 776 | True | DCEI077 | 11 |
| 100 | 1073 | True | DCEI078 | 17 |
| 100 | 1375 | True | DCEI079 | 26 |
| 100 | 1333 | True | DCEI080 | 5 |
| 100 | 935 | True | DCEI081 | 27 |
| 100 | 8169 | True | DCEI082 | 29 |
| 100 | 691 | True | DCEI083 | 1 |
| 100 | 777 | True | DCEI084 | 13 |
| 100 | 1418 | True | DCEI085 | 28 |
| 100 | 1376 | True | DCEI086 | 9 |
| 100 | 1389 | True | DCEI087 | 7 |
| 100 | 656 | True | DCEI088 | 14 |
| 100 | 1751 | True | DCEI089 | 20 |
| 100 | 932 | True | DCEI090 | 11 |
| 100 | 1507 | True | DCEI091 | 19 |
| 100 | 2724 | True | DCEI092 | 22 |
| 100 | 1531 | True | DCEI093 | 5 |
| 100 | 991 | True | DCEI094 | 19 |
| 100 | 607 | True | DCEI095 | 10 |
| 100 | 1718 | True | DCEI096 | 12 |
| 100 | 936 | True | DCEI097 | 16 |
| 100 | 2722 | True | DCEI098 | 30 |
| 100 | 920 | True | DCEI099 | 7 |
| 100 | 1342 | True | DCEI100 | 29 |
| 100 | 1042 | True | DCEI101 | 2 |
| 100 | 1584 | True | DCEI102 | 14 |
| 100 | 2834 | True | DCEI103 | 17 |
| 100 | 1499 | True | DCEI104 | 4 |
| 100 | 1525 | True | DCEI105 | 1 |
| 100 | 1892 | True | DCEI106 | 13 |
| 100 | 1128 | True | DCEI107 | 26 |
| 100 | 1228 | True | DCEI108 | 25 |
| 100 | 1638 | True | DCEI109 | 27 |
| 100 | 613 | True | DCEI110 | 9 |
| 100 | 2794 | True | DCEI111 | 28 |
| 100 | 1465 | True | DCEI112 | 3 |
| 100 | 2696 | True | DCEI113 | 8 |
| 100 | 1601 | True | DCEI114 | 18 |
| 100 | 917 | True | DCEI115 | 25 |
| 100 | 1663 | True | DCEI116 | 26 |
| 100 | 530 | True | DCEI117 | 7 |
| 100 | 1260 | True | DCEI118 | 14 |
| 100 | 1673 | True | DCEI119 | 9 |
| 100 | 604 | True | DCEI120 | 27 |
| 100 | 1232 | True | DCEI121 | 8 |
| 100 | 1272 | False | DCEI122 | 28 |
| 99 | 1434 | False | DCEI123 | 15 |
| 99 | 2215 | False | DCEI124 | 29 |
| 99 | 1788 | False | DCEI125 | 4 |
| 96 | 1035 | False | DCEI126 | 18 |
| 95 | 3797 | False | DCEI127 | 2 |
| 95 | 426728 | False | DCEI128 | 9 |
| 95 | 711 | False | DCEI129 | 5 |
| 95 | 1292750 | False | DCEI130 | 23 |
| 95 | 644 | False | DCEI131 | 21 |
| 95 | 1623 | False | DCEI132 | 2 |
| 95 | 539 | False | DCEI133 | 23 |
| 94 | 453 | False | DCEI134 | 18 |
| 94 | 2111 | False | DCEI135 | 8 |
| 94 | 384 | False | DCEI136 | 24 |
| 94 | 3206 | False | DCEI137 | 19 |
| 93 | 92012 | False | DCEI138 | 18 |
| 93 | 922 | False | DCEI139 | Not allocated |
| 93 | 697563 | False | DCEI140 | 22 |
| 93 | 381 | False | DCEI141 | Not allocated |
| 93 | 126 | False | DCEI142 | Not allocated |
| 93 | 24642 | False | DCEI143 | Not allocated |
| 93 | 283 | False | DCEI144 | 15 |
| 93 | 712 | False | DCEI145 | 21 |
| 93 | 1090 | False | DCEI146 | Not allocated |
| 93 | 511 | False | DCEI147 | Not allocated |
| 93 | 2150 | False | DCEI148 | Not allocated |
| 4 | 153 | False | DCEI149 | Not allocated |
| 4 | 241 | False | DCEI150 | Not allocated |
| 3 | 266 | False | DCEI151 | Not allocated |
| 3 | 2271 | False | DCEI152 | Not allocated |
| 3 | 909 | False | DCEI153 | Not allocated |
| 3 | 122 | False | DCEI154 | Not allocated |
| 3 | 212 | False | DCEI155 | Not allocated |
| 3 | 249 | False | DCEI156 | Not allocated |
| 3 | 209 | False | DCEI157 | Not allocated |
| 3 | 114 | False | DCEI158 | Not allocated |
| 3 | 229 | False | DCEI159 | Not allocated |
| 3 | 186 | False | DCEI160 | Not allocated |
| 3 | 198 | False | DCEI161 | Not allocated |
| 3 | 140 | False | DCEI162 | Not allocated |
| 3 | 910 | False | DCEI163 | Not allocated |
| 2 | 63 | False | DCEI164 | Not allocated |
| 2 | 60 | False | DCEI165 | Not allocated |
| 2 | 116 | False | DCEI166 | Not allocated |
| 2 | 94 | False | DCEI167 | Not allocated |
| 2 | 130 | False | DCEI168 | Not allocated |
| 2 | 1298179 | False | DCEI169 | Not allocated |
| 2 | 198 | False | DCEI170 | Not allocated |
| 2 | 76 | False | DCEI171 | Not allocated |
| 2 | 77 | False | DCEI172 | Not allocated |
| 2 | 315 | False | DCEI173 | Not allocated |
| 2 | 2363 | False | DCEI174 | Not allocated |
| 2 | 1044 | False | DCEI175 | Not allocated |
| 2 | 57 | False | DCEI176 | Not allocated |
| 2 | 572 | False | DCEI177 | Not allocated |
| 2 | 228 | False | DCEI178 | Not allocated |
| 2 | 123 | False | DCEI179 | Not allocated |
| 2 | 156 | False | DCEI180 | Not allocated |
| 2 | 335 | False | DCEI181 | Not allocated |
| 1 | 14 | False | DCEI182 | Not allocated |
| 1 | 114 | False | DCEI183 | Not allocated |
| 1 | 52 | False | DCEI184 | Not allocated |
| 1 | 45 | False | DCEI185 | Not allocated |
| 1 | 979 | False | DCEI186 | Not allocated |
| 1 | 81 | False | DCEI187 | Not allocated |
| 1 | 47 | False | DCEI188 | Not allocated |
| 1 | 8468 | False | DCEI189 | Not allocated |
| 1 | 37 | False | DCEI190 | Not allocated |
| 1 | 73 | False | DCEI191 | Not allocated |
| 1 | 96 | False | DCEI192 | Not allocated |
| 1 | 181 | False | DCEI193 | Not allocated |
| 1 | 152 | False | DCEI194 | Not allocated |
| 1 | 92 | False | DCEI195 | Not allocated |
| 1 | 22234 | False | DCEI196 | Not allocated |
| 1 | 259 | False | DCEI197 | Not allocated |
| 1 | 108 | False | DCEI198 | Not allocated |
| 1 | 57 | False | DCEI199 | Not allocated |
| 1 | 98 | False | DCEI200 | Not allocated |
| 1 | 1612 | False | DCEI201 | Not allocated |
| 0 | 54 | False | DCEI202 | Not allocated |
| 0 | 13 | False | DCEI203 | Not allocated |
| 0 | 18 | False | DCEI204 | Not allocated |
| 0 | 9 | False | DCEI205 | Not allocated |
| 0 | 39 | False | DCEI206 | Not allocated |
| 0 | 184377 | False | DCEI207 | Not allocated |
| 0 | 9326 | False | DCEI208 | Not allocated |
| 0 | 38 | False | DCEI209 | Not allocated |
| 0 | 2948 | False | DCEI210 | Not allocated |
| 0 | 139 | False | DCEI211 | Not allocated |
| 0 | 59 | False | DCEI212 | Not allocated |
| 0 | 43 | False | DCEI213 | Not allocated |
| 0 | 26 | False | DCEI214 | Not allocated |
| 0 | 22 | False | DCEI215 | Not allocated |
| 0 | 5 | False | DCEI216 | Not allocated |
| 0 | 13 | False | DCEI217 | Not allocated |
| 0 | 805339 | False | DCEI218 | Not allocated |
| 0 | 17 | False | DCEI219 | Not allocated |
| 0 | 62 | False | DCEI220 | Not allocated |
| 0 | 51 | False | DCEI221 | Not allocated |
| 0 | 6 | False | DCEI222 | Not allocated |
| 0 | 161 | False | DCEI223 | Not allocated |
| 0 | 49 | False | DCEI224 | Not allocated |
| 0 | 26 | False | DCEI225 | Not allocated |
| 0 | 30 | False | DCEI226 | Not allocated |
| 0 | 19 | False | DCEI227 | Not allocated |
| 0 | 89 | False | DCEI228 | Not allocated |
| 0 | 51 | False | DCEI229 | Not allocated |
| 0 | 20 | False | DCEI230 | Not allocated |
| 0 | 3 | False | DCEI231 | Not allocated |
| 0 | 39 | False | DCEI232 | Not allocated |
| 0 | 14 | False | DCEI233 | Not allocated |
| 0 | 199 | False | DCEI234 | Not allocated |
| 0 | 28 | False | DCEI235 | Not allocated |
| 0 | 24 | False | DCEI236 | Not allocated |
| 0 | 174 | False | DCEI237 | Not allocated |
| 0 | 393 | False | DCEI238 | Not allocated |
| 0 | 23 | False | DCEI239 | Not allocated |
| 0 | 6 | False | DCEI240 | Not allocated |
| 0 | 44 | False | DCEI241 | Not allocated |
| 0 | 14 | False | DCEI242 | Not allocated |
| 0 | 347854 | False | DCEI243 | Not allocated |
| 0 | 65 | False | DCEI244 | Not allocated |
| 0 | 174 | False | DCEI245 | Not allocated |
| 0 | 1013034 | False | DCEI246 | Not allocated |
| 0 | 564908 | False | DCEI247 | Not allocated |
| 0 | 74 | False | DCEI248 | Not allocated |
