# Supplementary material for: Patient preferences for Remote cochlear implant management: A discrete choice experiment
Source: PLoS One. 2025 Jun 3;20(6):e0320421. doi: 10.1371/journal.pone.0320421 (PMC12133006; doi:10.1371/journal.pone.0320421)
Supplement: S1 File — (DOCX) [file pone.0320421.s006.docx]

**DCE Survey:** Example of online survey showing information provided on Remote Check, one block of discrete choice experiment options, and additional questions on Remote Check.

**COCHLEAR IMPLANT CARE OPTIONS WITH REMOTE CHECK**

**Background:**

Cochlear implant (CI) clinics generally provide CI care with a fixed schedule of appointments, with little opportunity for personalisation. As a result, CI users may;

- attend appointments that provide little benefit but use up significant time and money for the CI user and the clinic OR
- choose to miss scheduled appointments if they are managing well, or are concerned about COVID risks and costs associated with attending appointments

Regular CI review helps audiologists detect a drop in hearing that may have gone unnoticed by the CI user.

Cochlear Ltd recently released the “Remote Check” add-onto the Nucleus Smart App that allows CI users to test and review some aspects of their CI function from home.

This questionnaire is designed to identify your preferences for how such a service could be provided, or if it is desired at all.

Please click on the link to read the Participant Information Form before completing the survey. If you have any further questions concerning this research project please do not hesitate to contact me.

***Please note if you have already completed this survey, please do not complete it again.***

**Consent Statement**

I have read the information provided and my questions, if I have any, have been answered to my satisfaction. I agree to participate in this research project, realising that I may withdraw at any time without reason and that this will not affect my relationship with my Hearing Clinic. However, I do understand that due to the anonymous nature of the survey, it will not be possible to remove data if I decide to withdraw from the study after the survey responses have been submitted.

**🔾 Yes 🔾 No**

**What is Remote Check?**

***What is Remote Check?***

Remote Check is add-on to the Nucleus Smart App, which you may use already to control your sound processor volume, programs etc.

- Your audiologist enrols you in Remote Check
- Once enrolled, you are notified that you have a test scheduled to complete through the Nucleus Smart App

A Remote Check Review has several features (described in the next couple of pages)…

***A Questionnaire:***

- asks about your general hearing, pain, discomfort or other issues you are having with your implant
- provides standard maintenance reminders (e.g. microphone cover changes)
- provides a place to indicate to you clinician if you need training or information on specific areas relating to your implant

***Photographs:***

- take and upload photos of your implant site through the Nucleus Smart App
- these can be reviewed later for any medical issues.

***A hearing test through your implant:***

- sounds are sent directly from your phone to your sound processor
- you indicate in the app whether or not you can hear them by moving the circle left or right

*Research has shown this test to be more accurate than the aided hearing tests performed in clinic.*

***A speech test through your implant:***

- groups of numbers are presented in background noise
- you type in which numbers (from 1-10) you have heard
- the noise gets louder or softer depending on the accuracy of your response

*Research has been shown this test is suitable for CI users and levels are stable over time.*

***An impedance test:***

- checks the function of your electrodes, like what happens when you are connected to the implant software in the clinic

*Research has shown impedance results from Remote Check are equivalent to impedance results obtained in the clinic.*

Your audiologist can choose to include any, or all, of these features for your remote check review.

When you finish your Remote Check review, you results are submitted through the Nucleus Smart App.

***What happens when you finish your Remote Check test?***

- Your results are stored securely on a remote server that is only accessible to staff from your implant clinic
- The person who reviews your Remote Check test can see the results of your previous Remote Check Test
- Any results that are different to the previous test are highlighted in red for close review by staff
- You are notified of the results of your test through the App, or another way as preferred

***What happens next?***

- Depending on the Remote Check review results there may be:
  - no further action
  - a follow up Remote Check review
  - or a clinic appointment
- Remote Check reviews can be scheduled at regular time points much like your regular clinical appointments

**I understand how a Remote Check review works.**

If you have additional questions about Remote Check, please contact [email address provided]

**🔾 Yes 🔾 No**

**General Demographics**

What is your age? (years). *Please type in a number between 18 and 120*

What is your gender? *[Drop down menu]*

What is your total household income per year? *[Drop down menu]*

**Scenarios**

You will be presented three different scenarios regarding management of your cochlear implant. These have been suggested as possible situations in which you could use Remote Check. There are several options about the care that you could receive when using Remote Check in these scenarios. These differ between **who** **reviews your Remote Check**, and **how** and **when** **your Remote Check is reviewed** and **how** **you receive your Remote Check feedback.**

*NB: Each scenario will be presented 4 times with different choice options each time.*

For each question below, please choose your preferred option between Option A and Option B.

**[BLOCK 01 shown as example]**

**Scenario 1a**

Imagine you are having a problem with your cochlear implant and it is suggested that you could complete a Remote Check test to test your cochlear implant function at home instead of waiting for a clinical appointment. You are offered two options, described below. If you had to choose between them, which would you pick?

|  | OPTION A |  | OPTION B |
| --- | --- | --- | --- |
| **After I submit my Remote Check my test will be reviewed by…** | my regular implant audiologist |  | trained administration staff who will refer to an audiologist if the test indicates a problem |
| **After I submit my Remote Check test, I will be sent…** | a detailed comparison of my hearing and speech tests with my previous results |  | a response only if the test indicates there was a problem |
| **Timing wise, I will get my Remote Check test results…** | within a week |  | the next day |
| **Remote Check test results will be provided to me by…** | a notification through the Nucleus Smart App |  | A face-to-face appointment with an audiologist |
| **Remote Check will cost me…** | $120 annual fee for an  unlimited number of checks |  | $40 annual fee for an  unlimited number of checks |
|  | **🔾 OPTION A** |  | **🔾 OPTION B** |

**Scenario 1b**

Imagine you are having a problem with your cochlear implant and it is suggested that you could complete a Remote Check test to test your cochlear implant function at home instead of waiting for a clinical appointment. You are offered two options, described below. If you had to choose between them, which would you pick?

|  | OPTION A |  | OPTION B |
| --- | --- | --- | --- |
| **After I submit my Remote Check my test will be reviewed by…** | any trained audiologist;  I don’t mind whether it is my  regular audiologist or not |  | my regular implant audiologist |
| **After I submit my Remote Check test, I will be sent…** | a response only if the test indicates there was a problem |  | a detailed comparison of my hearing and speech tests with my previous results |
| **Timing wise, I will get my Remote Check test results…** | within 2 weeks |  | the next day |
| **Remote Check test results will be provided to me by…** | a videoconference or phone call from my audiologist |  | a notification through the Nucleus Smart App |
| **Remote Check will cost me…** | $10 each time my Remote Check test results are reviewed by the clinic |  | $30 each time my Remote Check test results are reviewed by the clinic |
|  | **🔾 OPTION A** |  | **🔾 OPTION B** |

**Scenario 1c**

Imagine you are having a problem with your cochlear implant and it is suggested that you could complete a Remote Check test to test your cochlear implant function at home instead of waiting for a clinical appointment. You are offered two options, described below. If you had to choose between them, which would you pick?

|  | OPTION A |  | OPTION B |
| --- | --- | --- | --- |
| **After I submit my Remote Check my test will be reviewed by…** | trained administration staff who will refer to an audiologist if the test indicates a problem |  | my regular implant audiologist |
| **After I submit my Remote Check test, I will be sent…** | a response only if the test indicates there was a problem |  | a response to indicate whether the  overall results were good or bad  and what to do next |
| **Timing wise, I will get my Remote Check test results…** | the next day |  | within a week |
| **Remote Check test results will be provided to me by…** | A face-to-face appointment with an audiologist |  | a report emailed to me |
| **Remote Check will cost me…** | $30 each time my Remote Check test results are reviewed by the clinic |  | $120 annual fee for an  unlimited number of checks |
|  | **🔾 OPTION A** |  | **🔾 OPTION B** |

**Scenario 1d**

Imagine you are having a problem with your cochlear implant and it is suggested that you could complete a Remote Check test to test your cochlear implant function at home instead of waiting for a clinical appointment. You are offered two options, described below. If you had to choose between them, which would you pick?

|  | OPTION A |  | OPTION B |
| --- | --- | --- | --- |
| **After I submit my Remote Check my test will be reviewed by…** | any trained audiologist;  I don’t mind whether it is my  regular audiologist or not |  | my regular implant audiologist |
| **After I submit my Remote Check test, I will be sent…** | a response only if the test indicates there was a problem |  | a detailed comparison of my hearing and speech tests with my previous results |
| **Timing wise, I will get my Remote Check test results…** | within a week |  | within 2 weeks |
| **Remote Check test results will be provided to me by…** | a notification through the Nucleus Smart App |  | a videoconference or phone call from my audiologist |
| **Remote Check will cost me…** | $120 annual fee for an  unlimited number of checks |  | $30 each time my Remote Check test results are reviewed by the clinic |
|  | **🔾 OPTION A** |  | **🔾 OPTION B** |

**Scenario 2a**

Imagine you are due for your yearly cochlear implant review and it is suggested that you complete a Remote Check test of your cochlear implant function at home. An optional, short "in-clinic" appointment is available after your Remote Check if it is required. You are offered two options, options for your Remote Check Review, described below. If you had to choose between them, which would you pick?

|  | OPTION A |  | OPTION B |
| --- | --- | --- | --- |
| **After I submit my Remote Check my test will be reviewed by…** | trained administration staff who will refer to an audiologist if the test indicates a problem |  | any trained audiologist;  I don’t mind whether it is my  regular audiologist or not |
| **After I submit my Remote Check test, I will be sent…** | a response to indicate whether the  overall results were good or bad  and what to do next |  | a detailed comparison of my hearing and speech tests with my previous results |
| **Timing wise, I will get my Remote Check test results…** | The next day |  | within a week |
| **Remote Check test results will be provided to me by…** | a report emailed to me |  | a videoconference or phone call from my audiologist |
| **Remote Check will cost me…** | $120 annual fee for an  unlimited number of checks |  | $30 each time my Remote Check test results are reviewed by the clinic |
|  | **🔾 OPTION A** |  | **🔾 OPTION B** |

**Scenario 2b**

Imagine you are due for your yearly cochlear implant review and it is suggested that you complete a Remote Check test of your cochlear implant function at home. An optional, short "in-clinic" appointment is available after your Remote Check if it is required. You are offered two options, options for your Remote Check Review, described below. If you had to choose between them, which would you pick?

|  | OPTION A |  | OPTION B |
| --- | --- | --- | --- |
| **After I submit my Remote Check my test will be reviewed by…** | any trained audiologist;  I don’t mind whether it is my  regular audiologist or not |  | my regular implant audiologist |
| **After I submit my Remote Check test, I will be sent…** | a response to indicate whether the  overall results were good or bad  and what to do next |  | a detailed comparison of my hearing and speech tests with my previous results |
| **Timing wise, I will get my Remote Check test results…** | within 2 weeks |  | the next day |
| **Remote Check test results will be provided to me by…** | a face-to-face appointment with an audiologist |  | a notification through the Nucleus Smart App |
| **Remote Check will cost me…** | $40 annual fee for an  unlimited number of checks |  | $30 each time my Remote Check test results are reviewed by the clinic |
|  | **🔾 OPTION A** |  | **🔾 OPTION B** |

**Scenario 2c**

Imagine you are due for your yearly cochlear implant review and it is suggested that you complete a Remote Check test of your cochlear implant function at home. An optional, short "in-clinic" appointment is available after your Remote Check if it is required. You are offered two options, options for your Remote Check Review, described below. If you had to choose between them, which would you pick?

|  | OPTION A |  | OPTION B |
| --- | --- | --- | --- |
| **After I submit my Remote Check my test will be reviewed by…** | my regular implant audiologist |  | any trained audiologist;  I don’t mind whether it is my  regular audiologist or not |
| **After I submit my Remote Check test, I will be sent…** | a response to indicate whether the  overall results were good or bad  and what to do next |  | a detailed comparison of my hearing and speech tests with my previous results |
| **Timing wise, I will get my Remote Check test results…** | the next day |  | within a week |
| **Remote Check test results will be provided to me by…** | a videoconference or phone call from my audiologist |  | a face-to-face appointment with an audiologist |
| **Remote Check will  cost me…** | $120 annual fee for an  unlimited number of checks |  | $10 each time my Remote Check test results are reviewed by the clinic |
|  | **🔾 OPTION A** |  | **🔾 OPTION B** |

**Scenario 2d**

Imagine you are due for your yearly cochlear implant review and it is suggested that you complete a Remote Check test of your cochlear implant function at home. An optional, short "in-clinic" appointment is available after your Remote Check if it is required. You are offered two options, options for your Remote Check Review, described below. If you had to choose between them, which would you pick?

|  | OPTION A |  | OPTION B |
| --- | --- | --- | --- |
| **After I submit my Remote Check my test will be reviewed by…** | any trained audiologist;  I don’t mind whether it is my  regular audiologist or not |  | my regular implant audiologist |
| **After I submit my Remote Check test, I will be sent…** | a detailed comparison of my hearing and speech tests with my previous results |  | a response to indicate whether the  overall results were good or bad  and what to do next |
| **Timing wise, I will get my Remote Check test results…** | within a week |  | within 2 weeks |
| **Remote Check test results will be provided to me by…** | a notification through the Nucleus Smart App |  | a report emailed to me |
| **Remote Check will  cost me…** | $120 annual fee for an  unlimited number of checks |  | $10 each time my Remote Check test results are reviewed by the clinic |
|  | **🔾 OPTION A** |  | **🔾 OPTION B** |

**Scenario 3a:**

Imagine you have recently got your cochlear implant. You aren’t due for another clinical appointment for a while, or you can’t make it to your next appointment, but you would like reassurance that things are progressing between appointments. You are offered a Remote Check test with two options, described below. If you had to choose between them, which would you pick?

|  | OPTION A |  | OPTION B |
| --- | --- | --- | --- |
| **After I submit my Remote Check my test will be reviewed by…** | any trained audiologist;  I don’t mind whether it is my  regular audiologist or not |  | trained administration staff who will refer to an audiologist if the test indicates a problem |
| **After I submit my Remote Check test, I will be sent…** | a response to indicate whether the  overall results were good or bad  and what to do next |  | a detailed comparison of my hearing and speech tests with my previous results |
| **Timing wise, I will get my Remote Check test results…** | within 2 weeks |  | the next day |
| **Remote Check test results will be provided to me by…** | a face-to-face appointment with an audiologist |  | a report emailed to me |
| **Remote Check will  cost me…** | $120 annual fee for an  unlimited number of checks |  | $10 each time my Remote Check test results are reviewed by the clinic |
|  | **🔾 OPTION A** |  | **🔾 OPTION B** |

**Scenario 3b:**

Imagine you have recently got your cochlear implant. You aren’t due for another clinical appointment for a while, or you can’t make it to your next appointment, but you would like reassurance that things are progressing between appointments. You are offered a Remote Check test with two options, described below. If you had to choose between them, which would you pick?

|  | OPTION A |  | OPTION B |
| --- | --- | --- | --- |
| **After I submit my Remote Check my test will be reviewed by…** | my regular implant audiologist |  | any trained audiologist;  I don’t mind whether it is my  regular audiologist or not |
| **After I submit my Remote Check test, I will be sent…** | a response only if the test indicates there was a problem |  | a detailed comparison of my hearing and speech tests with my previous results |
| **Timing wise, I will get my Remote Check test results…** | the next day |  | within a week |
| **Remote Check test results will be provided to me by…** | a face-to-face appointment with an audiologist |  | a report emailed to me |
| **Remote Check will  cost me…** | $30 each time my Remote Check test results are reviewed by the clinic |  | $120 annual fee for an  unlimited number of checks |
|  | **🔾 OPTION A** |  | **🔾 OPTION B** |

**Scenario 3c:**

Imagine you have recently got your cochlear implant. You aren’t due for another clinical appointment for a while, or you can’t make it to your next appointment, but you would like reassurance that things are progressing between appointments. You are offered a Remote Check test with two options, described below. If you had to choose between them, which would you pick?

|  | OPTION A |  | OPTION B |
| --- | --- | --- | --- |
| **After I submit my Remote Check my test will be reviewed by…** | trained administration staff who will refer to an audiologist if the test indicates a problem |  | my regular implant audiologist |
| **After I submit my Remote Check test, I will be sent…** | a response to indicate whether the overall results were good or bad and what to do next |  | a response only if the test indicates there was a problem |
| **Timing wise, I will get my Remote Check test results…** | within a week |  | within 2 weeks |
| **Remote Check test results will be provided to me by…** | a report emailed to me |  | a notification through the Nucleus Smart App |
| **Remote Check will  cost me…** | $30 each time my Remote Check test results are reviewed by the clinic |  | $10 each time my Remote Check test results are reviewed by the clinic |
|  | **🔾 OPTION A** |  | **🔾 OPTION B** |

**Scenario 3d:**

Imagine you have recently got your cochlear implant. You aren’t due for another clinical appointment for a while, or you can’t make it to your next appointment, but you would like reassurance that things are progressing between appointments. You are offered a Remote Check test with two options, described below. If you had to choose between them, which would you pick?

|  | OPTION A |  | OPTION B |
| --- | --- | --- | --- |
| **After I submit my Remote Check my test will be reviewed by…** | any trained audiologist;  I don’t mind whether it is my  regular audiologist or not |  | trained administration staff who will refer to an audiologist if the test indicates a problem |
| **After I submit my Remote Check test, I will be sent…** | a detailed comparison of my hearing and speech tests with my previous results |  | a response to indicate whether the overall results were good or bad and what to do next |
| **Timing wise, I will get my Remote Check test results…** | within 2 weeks |  | within a week |
| **Remote Check test results will be provided to me by…** | a report emailed to me |  | a videoconference or phone call from my audiologist |
| **Remote Check will  cost me…** | $120 annual fee for an  unlimited number of checks |  | $40 annual fee for an  unlimited number of checks |
|  | **🔾 OPTION A** |  | **🔾 OPTION B** |

**Remote Check questions**

**1.** Based on my understanding of how Remote Check works, I would be comfortable using Remote Check in the following situations… (please indicate all responses that apply)

1. Troubleshooting if I am having a problem with my implant – Only attending the clinic if the problem can't be solved with remote check
2. Monitoring (while getting used to my cochlear implant in the first 6months after the) – keep the same number of in-clinic appointments as necessary
3. To complete the speech and hearing testing parts of my annual review appointment - come into the clinic for my annual mapping
4. Monitoring (once my hearing is stable - usually after 6-12 months after the implant) - only come into the clinic if there is a problem picked up with the testing (e.g. need mapping)
5. None of these. I want to be seen in the clinic for all my appointments

**2.** My experience with Remote Check could best be described as…

1. I had not heard about Remote Check before this survey
2. I had heard about Remote Check but I have not used it
3. I have used Remote Check before

**3.** Based on my knowledge of Remote Check, and my own cochlear implant experience, I think the best time to be told about Remote Check is:

1. When discussing which device to get prior to having the implant surgery
2. When the implant is switched on
3. A few weeks after the implant is switched on
4. After the implant fitting is stabilised and the CI user is comfortable with how the implant works (3–6 months after implantation)
5. When the implant management moves to annual review (12 months after implantation)
6. It doesn't matter

**4.** If you had been told about Remote Check before your cochlear implant surgery how much influence do you think this would have had on your decision about which brand of cochlear implant (e.g. Cochlear Ltd, Med-El, Advanced Bionics, Oticon Medical etc) to get?

1. Not at all influential
2. Slightly influential
3. Somewhat influential
4. Very influential
5. Extremely influential

**Nucleus Smart App questions**

1. My experience with the Nucleus Smart App (which I can use to control my sound processor) could best be described as…..
   1. I had not heard about the Nucleus Smart App
   2. I had heard about the Nucleus Smart App but I have not used it
   3. I have downloaded the Nucleus Smart App but I do not use it
   4. I use the Nucleus Smart App occasionally (less than once a week)
   5. I use the Nucleus Smart App regularly (once a week or more)
2. If I use the Nucleus Smart App (which I can use to control my sound processor) I would say that I am…. (please indicate the most appropriate option)
   1. not confident at all using it
   2. slightly confident at using it
   3. somewhat confident at using it
   4. fairly confident at using it
   5. completely confident at using it
   6. I don’t use the Nucleus Smart App

**General Cochlear Implant Questions**

1. I regularly have problems with my sound processor (outside parts of the implant)...
   1. Strongly Disagree
   2. Somewhat disagree
   3. Neither Agree nor Disagree
   4. Somewhat Agree
   5. Strongly Agree
2. I regularly have problems with my internal implant...
   1. Strongly Disagree
   2. Somewhat Disagree
   3. Neither Agree nor Disagree
   4. Somewhat Agree
   5. Strongly Agree
3. I am satisfied with the outcome of my cochlear implant/s
4. Strongly Disagree
5. Somewhat disagree
6. Neither Agree nor Disagree
7. Somewhat Agree
8. Strongly Agree

Please add an additional comment explaining your answer above if you feel further clarification is required.

1. My hearing device use is best described as (please indicate the most appropriate option):

| cochlear implant in one ear  normal/good hearing in the other ear, no hearing aid |  |
| --- | --- |
| cochlear implant in one ear  Poor/no hearing in the other ear, no hearing aid |  |
| cochlear implant in one ear  Hearing aid in the other ear which provides good hearing |  |
| cochlear implant in one ear  Hearing aid in the other ear but limited hearing |  |
| Cochlear implants in both ears |  |

1. I have been using my cochlear implant/s for….

|  | Left | Right |
| --- | --- | --- |
| Less than ½ a year |  |  |
| ½ a year to less than 1 year |  |  |
| 1 year to less than 2 years |  |  |
| 2 years to less than 5 years |  |  |
| 5 years to less than 10 years |  |  |
| 10 years to less than 20 years |  |  |
| More than 20 years |  |  |
| Not applicable |  |  |

1. How long does it generally take you to travel to your regular cochlear implant clinic (in hours)? (Numeric text response)
2. Please indicate your level of agreement with the following statement…. In general, I am easily able to make time to attend my cochlear implant appointments

| Strongly disagree |  |
| --- | --- |
| Disagree |  |
| Neither agree nor disagree |  |
| Agree |  |
| Strongly agree |  |

There are no further questions in the survey. 
 
The outcome will provide us with valuable information about how we might better personalise cochlear implant care in the future.  
 
If you wish to be advised of the outcome of the study, please provide your email address below:
